# Data description of “building age map, Vienna, around 1920”

**DOI:** 10.1016/j.dib.2022.107864

**Published:** 2022-01-23

**Authors:** Ferdinand Reimer, Ulrich Kral, Emre Can Sönmez, Friedrich Hauer, Severin Hohensinner, Hannah Wolfinger, Klara Stuppacher, Andreas Danzinger, Ingeborg Hengl, Lupina Prospero, Sarah Prunner, Helmut Rechberger

**Affiliations:** a Institute for Water Quality and Resource Management, Technische Universität Wien, Karlsplatz 13/226, Vienna 1040, Austria; b Unaffiliated, Vienna, Austria; c Institute of Urban Design and Landscape Architecture - Research Unit Urban Design, Technische Universität Wien, Karlsplatz 13/260, Vienna 1040, Austria; d Institute of Hydrobiology and Aquatic Ecosystem Management, University of Natural Resources and Life Sciences Vienna, Gregor-Mendel-Straße 33/DG, Vienna 1180, Austria

**Keywords:** Historic GIS, Building stock, Building period, Construction date, Digitalization

## Abstract

Building age maps inventory the construction dates of buildings. While many cities routinely map the construction dates of present building stocks, building age maps of the distant past are mostly not available. An exception is the building age map of Vienna around 1920. It covers about 80% of the building footprint area within the city boundary in 2020 and is available in analog format only. This impedes spatial analysis of the building stock in the past and the production of time-series data for the spatio-temporal analysis of building stock developments over the last 100 years. To create the digital map, we manually vectorized 80,640 building footprints from 134 historical map sheets and assigned construction dates (i) from the analog building age map by digitizing color-encoded thematic information and (2) from a historical building registry by matching building address. From the analysis of the generated dataset we infer that the total building footprint area was 2,279 hectares. The classification of the building footprint areas by construction date shows that 14% of the buildings were older and 63% were younger than 70 years. The remaining 23% lack construction period assignments due to missing data. The resulting dataset underwent technical quality checks and external data sources were used to validate the building counts, the building presence around 1920 and the construction dates of buildings. During course of validation, we critically discuss data quality and recommend improvements. We see a practical reuse value of the data for the spatio-temporal analysis of urban buildings stocks, which facilitates urban history research as well as resource and environmental management in the city of Vienna.

**Specifications Table**

**Table:** 

Subject	Geographical information system
Specific subject area	Building footprints and construction periods
Type of data	Geospatial dataset
How data were acquired	The creation of the geospatial dataset is based on desktop work. The data were acquired by vectorizing building footprints from analog historic maps and by retrieving construction dates from an analog thematic map on building periods and a city-wide statistic on construction dates.
Data format	Secondary data
Parameters for data collection	The spatial coverage is the city of Vienna, which is the capital of Austria (Latitude: 48° 12′ 30.56” N, 16° 22′ 19.49” E) [1]. The conditions for data collection consider the timestamp, the spatial accuracy, and the city-wide availability of information. We used data sources that represent the 1920 situation as best as possible, that allow to reconstruct the building footprints by location and geometry, and that provide best available data on construction dates.
Description of data collection	The data inputs were collected from data repositories and archives as detailed in the next section.
Data source location	Data inputs: • Dataset: City boundary 1920 [2] • Dataset: Urban district boundaries 1920 [2] • Analog map: Analog building age map 1920 [3] • Analog map: Historical urban district maps [4], [5], [6], [7], [8], [9], [10], [11], [12], [13], [14], [15], [16] • Analog map: Historical cadaster [17] • Analog map: Historical fire brigade map [18], [19], [20], [21], [22], [23], [24] • Dataset: City map 2018 [25] • Dataset: Addresses Locations Vienna 2019 [26] • Dataset: Administrative borders 2020 [27] • Dataset: Street graph 2020 [28] • Dataset: Building schematic of Vienna in the late 1920s [29] • Dataset: Landscape structure map 1912 [30] • Analog map: Urban sprawl map [31] Data input descriptions can be found in Table 2.
Data accessibility	Repository name: Zenodo Data identification number: 3715200 Direct URL to data: 10.5281/zenodo.3715200 Directory: Building age map, Vienna, around 1920

**Value of the Data**

• With respect to Vienna, city-wide GIS data with building footprints are available for the year 1829 [32] and for the years as of 1995 [33]. This article presents a new GIS data snapshot, capturing the building footprints at around 1920. The GIS data are enriched by the construction periods of the buildings at this time. We feel the dataset, briefly called BSM_1920, is useful for spatio-temporal analysis of Vienna's building stock in the context urban development, urban history, environmental history, and waste and resource management.
• The BSM_1920 dataset is valuable for urban historians, city planners, waste and resource managers and interested members of the public.
• The BSM_1920 dataset can be used in the following disciplines:
  ○ *Urban development:* Prior to the generation of this dataset, we developed a prototype model to identify demolition and construction dynamics from 1920 to 2018 [34]. During the prototype development, we reconstructed building footprints for 3% of the city area only. The BSM_1920 dataset allows us to analyze the housing dynamics across all urban districts and for the city as a whole.
  ○ *Buildings history:* The BSM_1920 dataset includes address data of the 1920s, which enables linkage with the building schematic of Vienna in the late 1920s [29],[35]. The linkage allows the number of floors, property area and the year of purchase to be assigned to the building footprints.
  ○ *Environmental history:* The BSM_1920 dataset can be used to analyze the relationship between urban development and the regulation or transformation of urban water streams and floodplains. This helps to analyze the transformation of land use from fluvially formed areas built up areas. With that, indications can be given for the soil conditions in historical building grounds.
  ○ *Waste and resource management:* The building footprint in the BSM_1920 database can be linked with material intensity databases [e.g. [36],[37] to estimate the historical material stock composition in terms of both quantity and quality. This allows to compare the historical and today's material reservoir, whereof data for 2013 are presented by Kleemann et al. [38]. Next, Džubur and Laner [39] estimated the historical building stock volume to predict wood waste flows in the future. At the time of investigation, evidence-based data on the area and volume of the building stock were not available. The BSM_1920 facilitates the characterization of the historical building stock and therefore improves the input data quality for waste outlooks in the city of Vienna.
  ○ *Data visualization:* The BSM_1920 can be used as a starting point for data visualization via interactive online maps, dashboards and storylines and to communicate the research findings to a broader audience.

## 1 Data Description

**Fig. 1:**
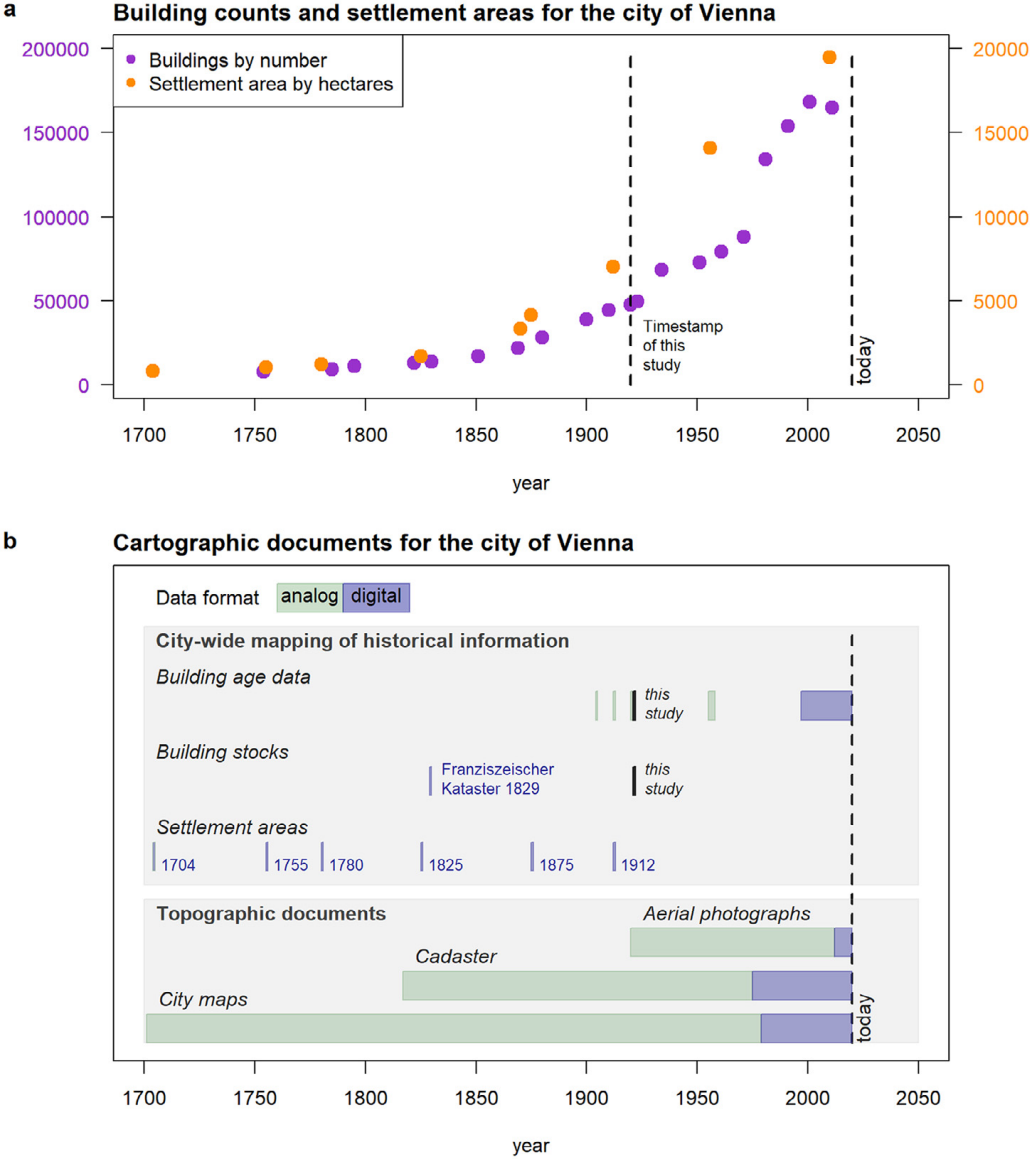
Background information for Vienna from the 18th to 21st century. Sub-part a: Development of building counts and settlement areas. Notes: Building numbers retrieved from Wien Geschichte Wiki https://www.geschichtewiki.wien.gv.at/Bev%C3%B6lkerung. Settlement area data documented in Zentrum für Umweltgeschichte (eds) [40] and provided by S. Hohensinner (https://orcid.org/0000–0002–3517–0259). Sub-part b: Cartographic documents, including the period of records on city maps, aerial photographs and in cadasters as well as geospatial datasets with reconstructions of historical city data. The generation of the building stock map of 1920 is documented by this article. *Notes:* “Analog” = Maps on paper or scans (non-machine-readable); “Digital” = Maps based on geographical information systems (machine-readable).

Urban archives traditionally provide rich information on the historical development of the building stock. The information is archived on cartographic documents, construction plans, pictures, statistics and registries. They are critical for far back looking approaches, but time-series data for spatial-explicit analysis of the building stocks is still hard to accomplish. This is also the case for the city of Vienna. Taking the period 1700–2020 into perspective shows that city maps, cadasters and aerial photographs picture the buildings in town (Fig. 1). The cartographic information became machine-readable with the establishment of computer systems and the continuous increase of data storage and computing power from 1980 onwards. Before that, the spatial information on buildings is only available on paper or diapositives (analog format) or on scan photographs and scan papers (digital but non-machine-readable format). The only city-wide GIS dataset with historical building footprints, at least to our knowledge, is the Franziszeische Kataster 1829 [32], which is a snapshot of the cadaster in 1829. Beside the building-specific mapping, researchers from the University of Natural Resources and Life Sciences, Vienna (BOKU), reconstructed the historical landscape structure and produced GIS datasets with the settlement areas in the years 1704, 1755, 1780, 1825, 1875 and 1912 [40].

An added value for time-series analysis of building stock development is given by the information on the age of buildings. In Vienna, the machine-readable building age maps started in the 1990s [41]. Previously, cartographic documents with building footprints and construction date labels were published on paper. Examples are the city map of 1904 and 1912 with fragmentary age labels across the city [42],[43], the building age map 1920 with nearly a full geographical coverage of buildings at this time [3], the city-wide mapping 1955–1959 with a full coverage of buildings and its building period [44], and the maps of 1969/72 for parts of the urban districts 15–18 [45], [46], [47], [48]. Regrettably, the maps of the distant past are not retrievable in machine-readable format. This prevents automatic data processing in view of spatio-temporal analysis of the Viennese building stock.

**Fig. 2:**
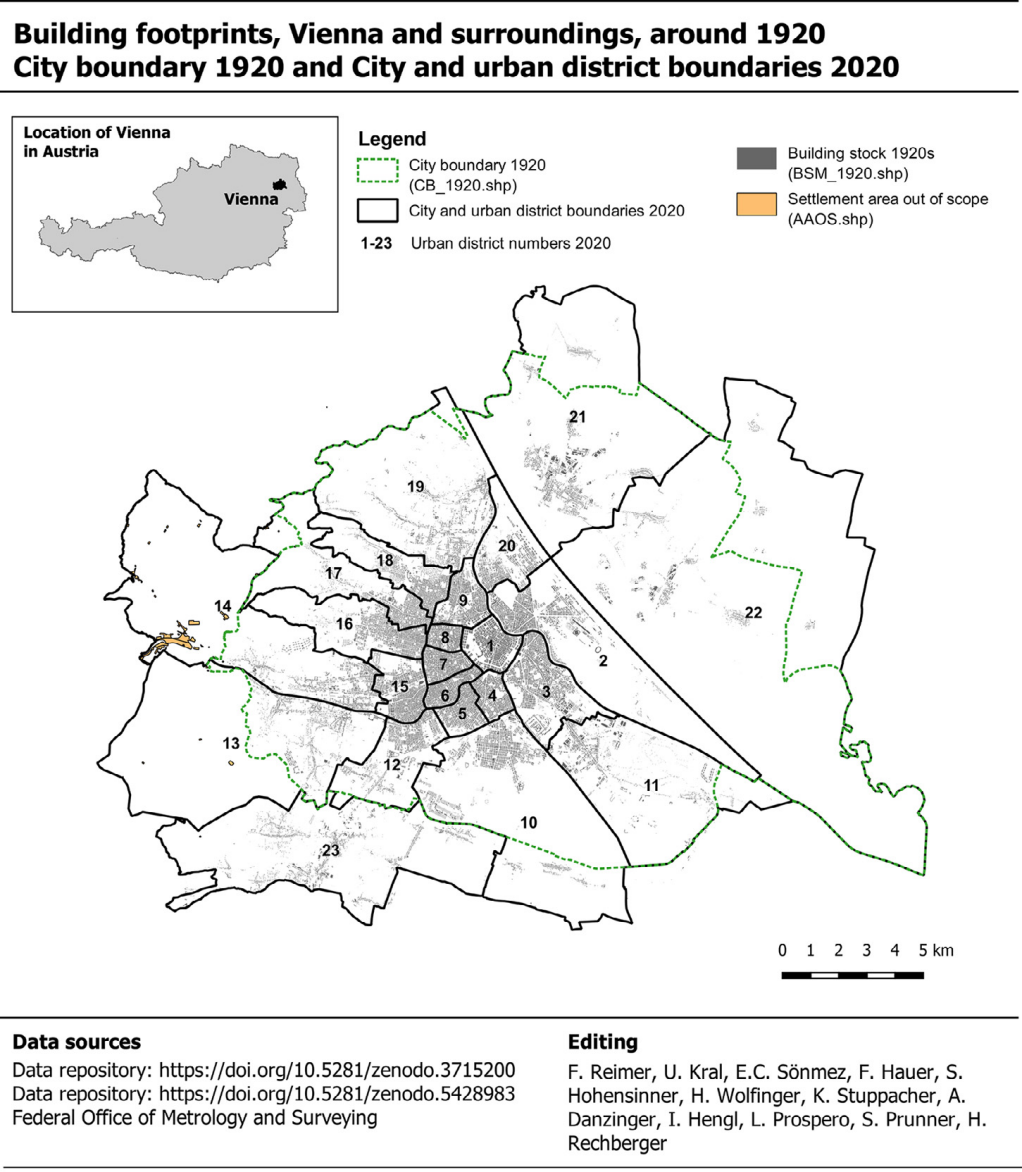
Mapping the GIS dataset BSM_1920 and the city boundary of 1920 and 2020. Data taken from Kral et al. [2], Federal Office of Metrology and Surveying [27], Reimer et al. [49].

In this work, we present a machine-readable geospatial dataset of the building footprints for the city of Vienna in the 1920s and assign the building period information to each building. The geographical coverage is defined by city area of 2020, which goes beyond the city area of 1920 and therefore includes the city's surroundings of 1920 too (Fig. 2). The timestamp of the building footprints is at around 1920 as well as construction periods assignments.

**Fig. 3:**
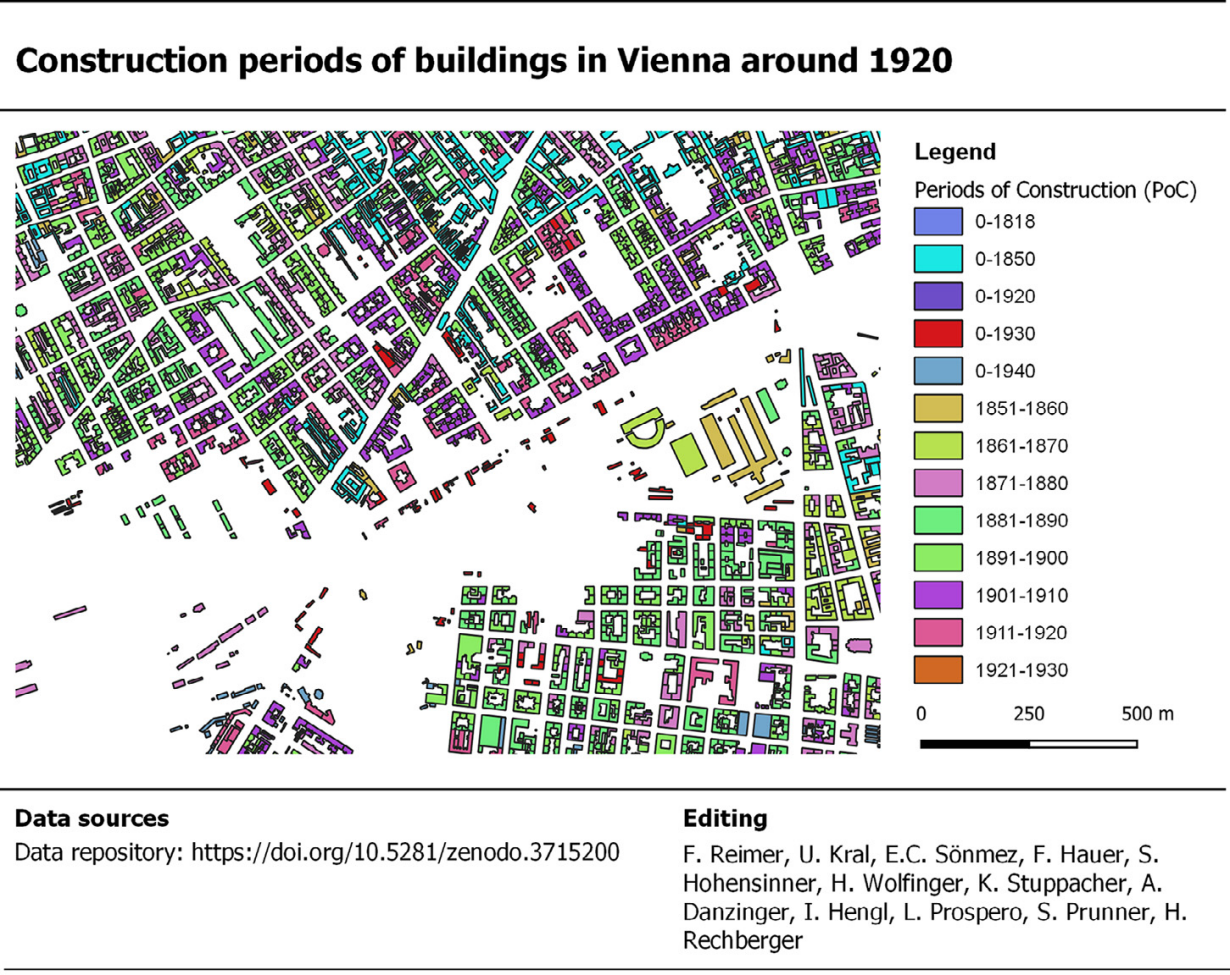
Construction periods of buildings in Vienna around 1920.

**Fig. 4:**
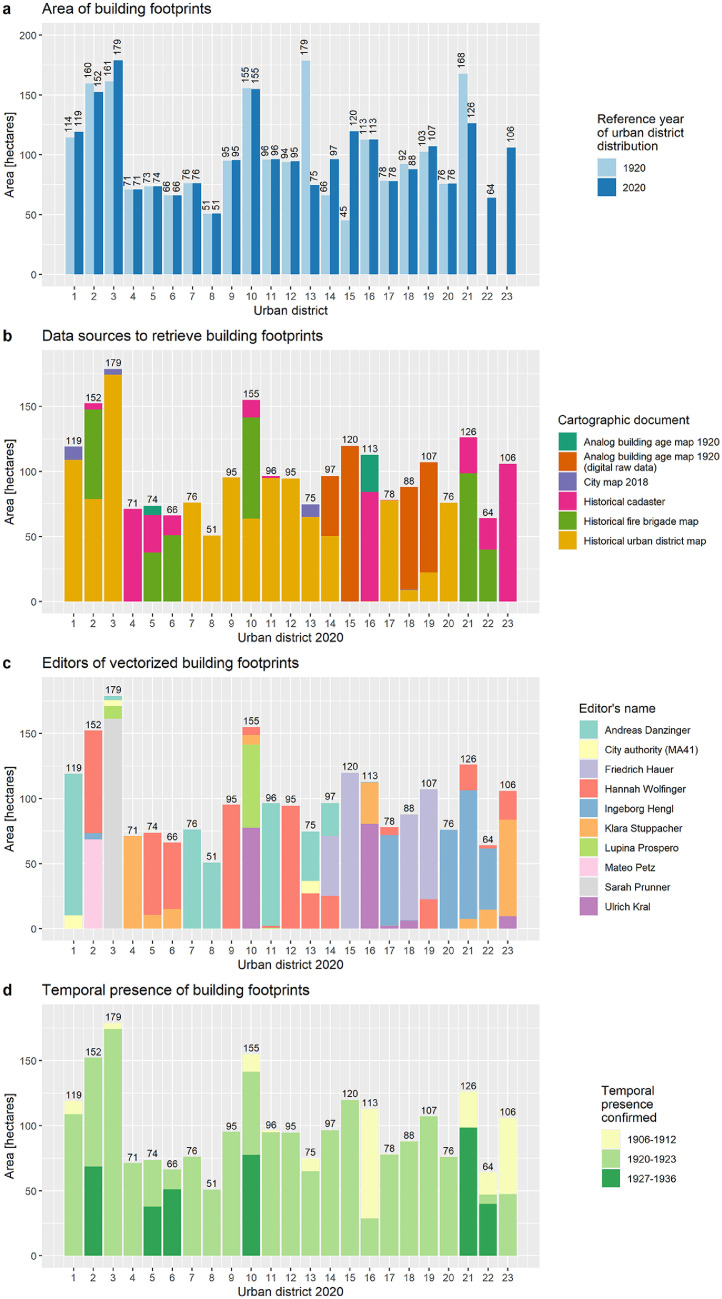
Summarized “Area” records grouped by “UD_1920” and “UD.2020”, “Polygon.source_id”, “Editor” and “TP_pub.date_period”. Sub-part a: Comparative building footprint areas. It is noted that the data records for urban districts numbers 22,23 in the reference year 1920 are not available because at this time the 22nd district was part of the 21st district and the area of the 23rd district was beyond the city boundary 1920 [c.f. [50]. It is also noted that the geographical coverage of urban districts differs between 1920 and 2020 because the boundaries were shifted to some extent. Sub-part b: Data sources to retrieve building footprints. The building footprints were retrieved from distinctive building stock maps because a single city-wide map with all building footprints around the year 1920 was not available. Sub-part c: Editors of vectorized building footprints. I In total, 8 persons digitized building footprints during our project lifetime between 2017 and 2020; Friedrich Hauer generated the building footprints before 2015 in view of the production of the ABAM_1920; the footprints of heritage buildings were provided by the city authority – department 41. Sub-part d: Temporal presence of building footprints by urban district. To generate the data for the plot, we clustered 20 unique data records from “TP_pub.date_year” into three periods. In a city-wide perspective, the total area of the building footprints is 2279 ha (100%), of which 1,679 ha (74%) are dated between 1920 and 1923, 373 ha (16%) are dated between 1927 and 1936 and 227 ha (10%) have a timestamp between 1906 and 1915. Note: For interpretation of the references to color in this figure legend, the reader is referred to the web version of this article.

**Fig. 5:**
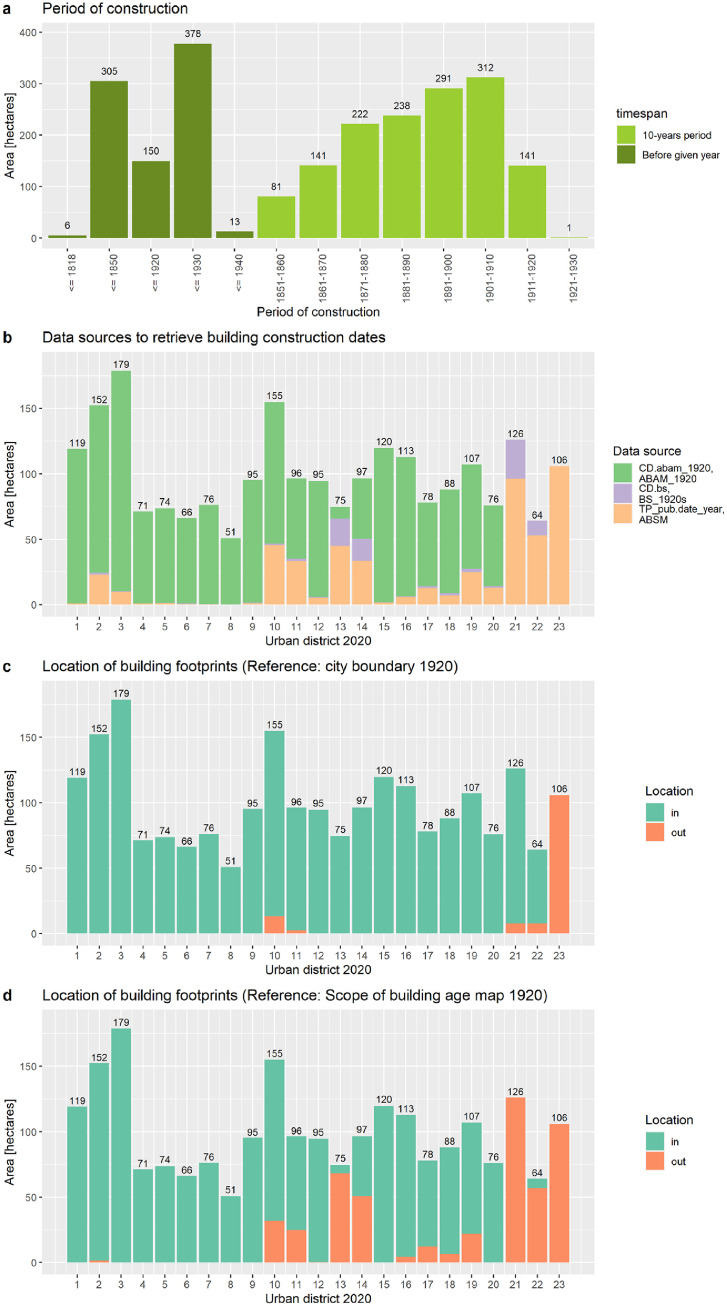
Summarized “Area” records grouped by “PoC”, “PoC.source_name”, “Location.city_limit_1920” and “Location.ABAM_1920”. Sub-part a: Periods of building construction. The building footprints with a 10-year PoC timespan represent 63% of the total area and those with a PoC before a given year represent 37%. Sub-part b: Data sources to retrieve building construction dates. The scope is the whole of Vienna. From a city-wide perspective, 73% of the building footprint areas received PoC assignments from the ABAM_1920 [3], 23% from the publication year of the analog building stock map and 4% from the construction year as given in BS_1920s [29]. Sub-part c: Location of building footprints with respect to the city limits 1920. From a city-wide perspective, the total building footprint area is 2,279 ha (100%), of which 2,142 ha (94%) are located within the city limits of 1920 and 137 ha (6%) are located outside – which belong to the city territory in 2020. Sub-part d: Location of building footprints with respect to the scope of the analog building age map 1920. It is noted that these number are based on building footprints within the city boundary of 1920. The ABAM_1920 covers 36% of the total city area at this time because the scope is limited to districts 1–20 without the outskirts and excludes the 21st district. In terms of building footprint areas, 78% of the total footprint area is covered by the ABAM_1920. The remaining 22% of building footprints were not captured and can be found at the outskirts of the city as well as in areas outside of the city boundary of 1920 but within the city boundary of 2020. Note: For interpretation of the references to color in this figure legend, the reader is referred to the web version of this article.

A city-wide map with the building footprints and the city boundaries of 1920 and 2020 is given in Fig. 2. The underlaying geospatial BSM_1920 dataset includes 80,640 polygon-features, which represent building footprints, and the corresponding attribute table includes 28 data fields (Table 1). One data field, namely the “PoC”, covers the period of construction data. It is noted that an urban section was randomly selected to demonstrate the appearance of PoC data on the map (Fig. 3). In addition, we selected 8 out of 28 data fields, grouped and summarized the data records and plotted them in Figs. 4 and 5. These figures demonstrate the documentation of key background data on the building level, such as the data sources for retrieving the geometry of the building footprint and its timestamp, and therefore facilitates transparency in data acquisition and processing.

**Table 1:** Description of dataset fields used in the building stock map 1920 and the corresponding attribute table, respectively. *Notes:* “ABAM_1920” = Analog building age map 1920 [3]. “BS_1920s” = Building schematic of Vienna in the late 1920s [29].

Field	Name	Description
ID	Identifier polygon-feature	A unique number for each polygon-feature.
ID.bs	Identifier building schematic	A unique number that corresponds with “ID” in the BS_1920s dataset.
Area	Area	The area of the polygon-feature in square meters.
UD.1920	Urban district number 1920	The number of the urban district in 1920.
UD.2020	Urban district number 2020	The number of the urban district in 2020.
Location.city_limit_1920	Location city limit	Location of the polygon-feature with respect to city limits 1920.
Location.ABAM_1920	Location ABAM_1920 scope	Location of the polygon-feature with respect to geographical coverage of ABAM_1920.
Polygon.source_id	Identifier polygon source	An identifier for the data source from which the polygon-feature was digitized or retrieved.
Polygon.source_name	Name of polygon source	Name of the data source from which the polygon-feature was digitized or retrieved.
Editor	Editor's name	Name of the person who vectorized the polygon or an institution that provided the polygon-feature.
CD.abam	Construction date ABAM_1920	Construction date retrieved from the ABAM_1920.
CD.bs	Construction date BS_1920s	Construction date retrieved from the BS_1920s dataset.
TP_pub.date	Temporal presence given by date	The temporal presence of building footprints based on the publication year of the analog historical building stock map, which shows the building footprints.
TP_pub.date_year	Temporal presence given by year	Temporal presence of building footprints given by year, retrieved and compiled from “TP_pub.date”.
TP_pub.date_period	Temporal presence given by periods	Temporal presence of building footprints given by period, retrieved and compiled from “TP_pub.date_year”.
PoC	Period of construction	The construction period of the building.
PoC.source_name	Source name of period of construction	The name of the data source from which we retrieved and compiled the period of construction “PoC”.
ID.Address.2019	Identifier for address point	Identifier for address point, retrieved from “Address Locations Vienna”.
Address.2019_reloc	Address point re-location	Documentation whether the location of the address point has been manually relocated or not.
Address.2019	Address in 2020	Address data for 2020, in particular the name of the traffic area and the orientation number.
Address.1920	Address in 1920	Address data for 1920, in particular the name of the traffic area and the orientation number.
Address_reviewed	Review status of address	Address data for 2020, reviewed by manual comparison with address labels in historical maps.
Address_revised	Revised address	Addresses under review (see “Address_reviewed”) were revised based on the address labels in historical building stock maps.
Polygon.source.Scale	Scale	Scales of building stock maps, which were used to retrieve the building footprints.
Location.usm	Spatial presence built-up area 1918	Spatial presence of the polygon-feature with respect to the built-up area in 1918.
Location.lsm	Spatial presence built-up area 1912	Spatial presence of the polygon-feature with respect to the built-up area in 1912.
Presence.conf	Spatial and temporal presence	Spatial and temporal presence of the building, whereas the spatial dimension refers to the presence in the built-up area of 1912 and 1918, respectively, and the temporal dimension refers to the publication date of analog historical building stock maps, which show the building footprints.
CD.bs_fit	Construction date fit	This data fields shows if the construction data “CD.bs” (assigned from the BS dataset) fits within the period of construction “PoC”.

## 2 Experimental Design, Materials and Methods

### 2.1 Data input

We used 13 distinctive data sources (Table 2) to create the dataset. All data sources are publicly accessible, whereas 7 can be retrieved online and 6 can be accessed by personal order only.

**Table 2:** Input data sources. *Notes:* “PDF” = file extension of Portable Document Format documents, “SHP” = file extension of shapefiles, “CSV” = file extension of Comma-Separated Value files, “TIF” = file extension of Tagged Image File.

Title	Description	Format	Access	Refs.
City boundary 1920	The dataset includes the city boundary of 1920. Acronym: CB_1920	SHP	Online	[2]
Urban district boundaries 1920	The dataset includes the urban district boundary of 1920. Acronym: UDB_1920	SHP	Online	[2]
Analog building age map 1920	A thematic map showing the construction period for the buildings in Vienna in the year 1920. Acronym: ABAM_1920	TIFF	Online	[3]
Historical urban district maps	The urban districts maps show buildings and their addresses. The map sheets do not cover the entire city. It is noted that the Municipal and Provincial Archives of Vienna provide two set of identical maps and indicate a timestamp of 1921 and 1930, respectively. The definition of the exact timestamp is challenging, because the maps show social houses that were constructed after 1925. This might be either, because cartographers in these days also mapped future development projects, as shown in city maps of 1904 and 1912 [42],[43], or because the maps were published later than 1921. However, we selected the timestamp of 1921 and feel that potential buildings (constructed 1921–30) have only a minor influence on the total built-up area.	TIFF	Online	[4], [5], [6], [7], [8], [9], [10], [11], [12], [13], [14], [15], [16]
Historical cadaster	The “Franziszeische Kataster” is the first complete Austrian cadaster, published between the 1810s and 1870s. Later on, the historical cadastral map sheets were manually updated to document, among other information, building footprints and plot boundaries.	TIFF	Personal order	[17]
Historical fire brigade map	The map sheets were published before 1920 and between 1926 and 1939. The map sheets cover only parts of Vienna at this time and show the buildings and their addresses as well as infrastructure (water pipes, hydrants) for the fire brigade.	TIFF	Online	[18], [19], [20], [21], [22], [23], [24]
City map 2018	Today's city map, also called area multipurpose map, includes georeferenced polygon-features for buildings.	SHP	Online	[25]
Addresses Locations Vienna 2019	The dataset includes address data, in particular the address designation (traffic area + orientation number), as well as the coordinates of the access address (property access) and the building address (location of the building entrances).	SHP	Online	[26]
Administrative borders 2020	The dataset covers the boundaries of the city, urban districts and cadastral communities in Vienna.	SHP	Online	[27]
Street graph 2020	The street graph includes the road network in the city of Vienna.	SHP	Online	[28]
Building schematic of Vienna in the late 1920s	The building schematic was a knowledge base for real estate and finance business. The schematic lists buildings and properties, respectively, and associated street name, building number, number of floors, property area, year of construction and year of purchase. Acronym: BS_1920s	CSV	Online	[29]
Landscape structure map 1912	The map includes water bodies, settlement areas, railway stations and different terrain types. Acronym: LSM	SHP	Personal order	[30]
Urban sprawl map	The map shows the city's extent in the years 1857, 1861, 1918, 1938 and 1957. Acronym: USM	TIFF	Online	[31]

### 2.2 Data processing

This section provides an overview on the approach to generate the datasets (Section 2.2.1) and a detailed description of the data processing steps by details on four data processing steps (Sections 2.2.2–2.2.4).

#### 2.2.1 Approach

**Fig. 6:**
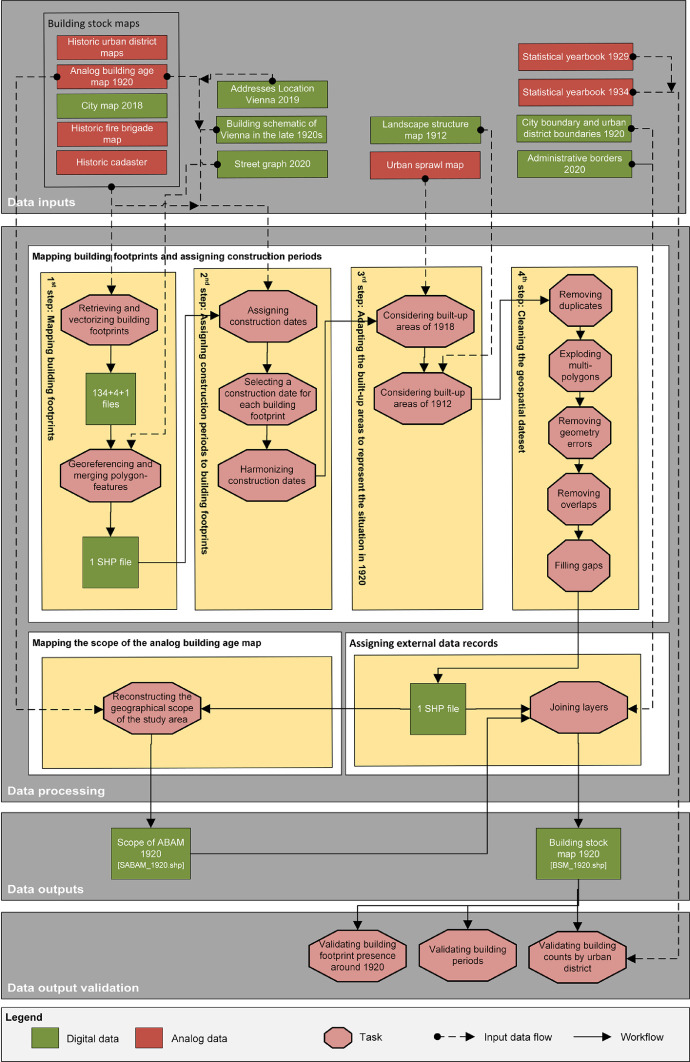
Workflow to generate and validate the final geospatial dataset with the scope of the analog building age map (SABAM.shp) as well as the building stock map (BSM_1920.shp) and the associated attribute table. Note 1: “Analog data” = Non-machine-readable data on paper or scans; “Digital data” = Machine-readable data. Note 2: For interpretation of the references to color in this figure legend, the reader is referred to the web version of this article.

**Fig. 7:**
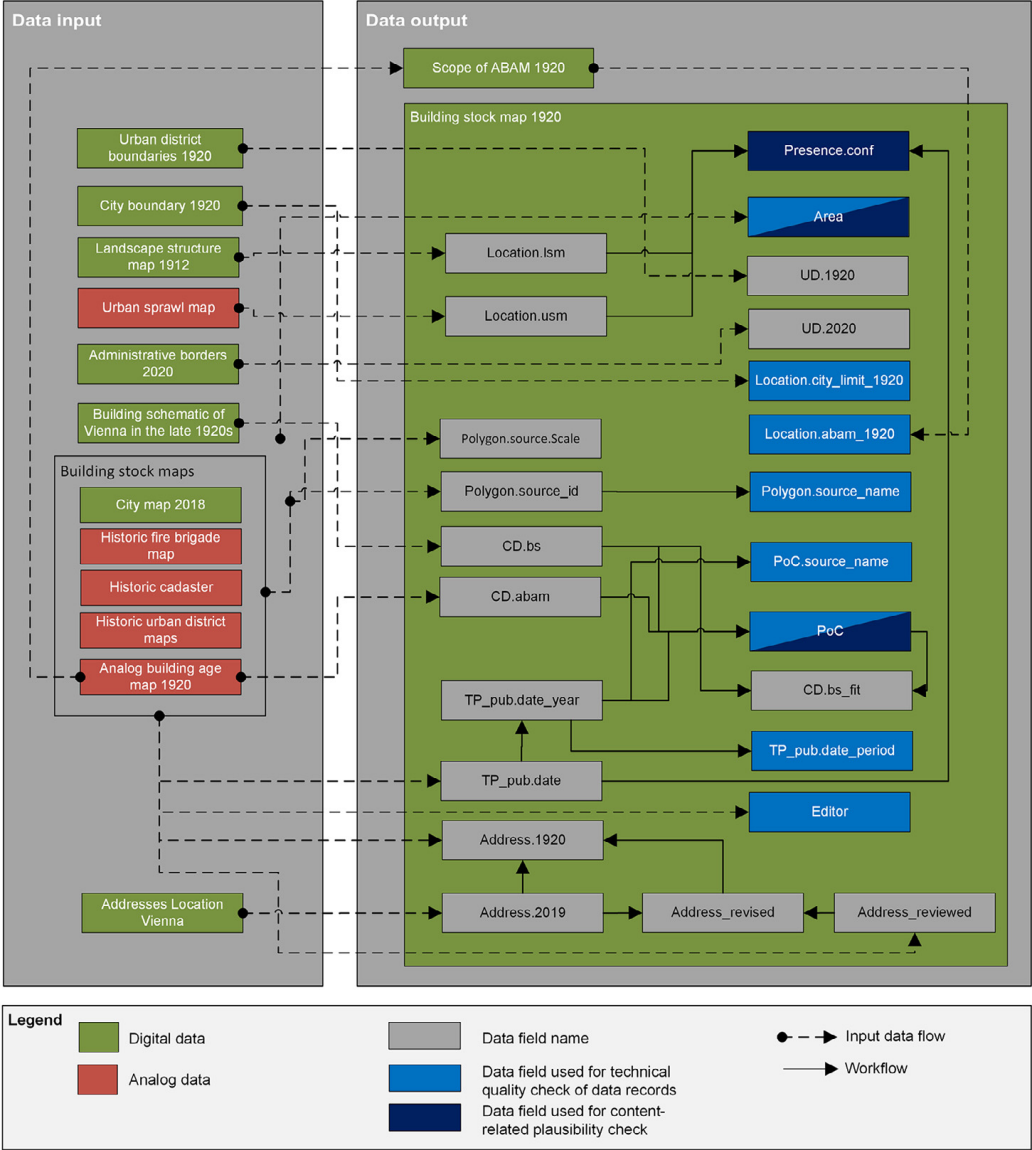
Relation of data fields in the attribute table of the building stock map 1920 [49] to their data inputs. The data fields are given as acronyms and described in Table 1 of this article. It is noted that the data fields with identifiers “ID”, “ID.bs” and “ID.Address.2019” as well as the “Address.2019_reloc” are excluded from this figure. *Note:* For interpretation of the references to color in this figure legend, the reader is referred to the web version of this article.

The workflow to generate the final dataset [49] covers four distinctive stages including data inputs, processing, outputs and validation (Fig. 6). The attribute table of the building stock map 1920 includes 28 data fields in total, of which 22 are shown with it's linkages to the data inputs and outputs in Fig. 7.

#### 2.2.2 Mapping building footprints and assigning construction periods

##### 2.2.2.1 1st step: Mapping building footprints

Our aim is to map the building footprints around 1920 within the city boundary of 2020.

**Fig. 8:**
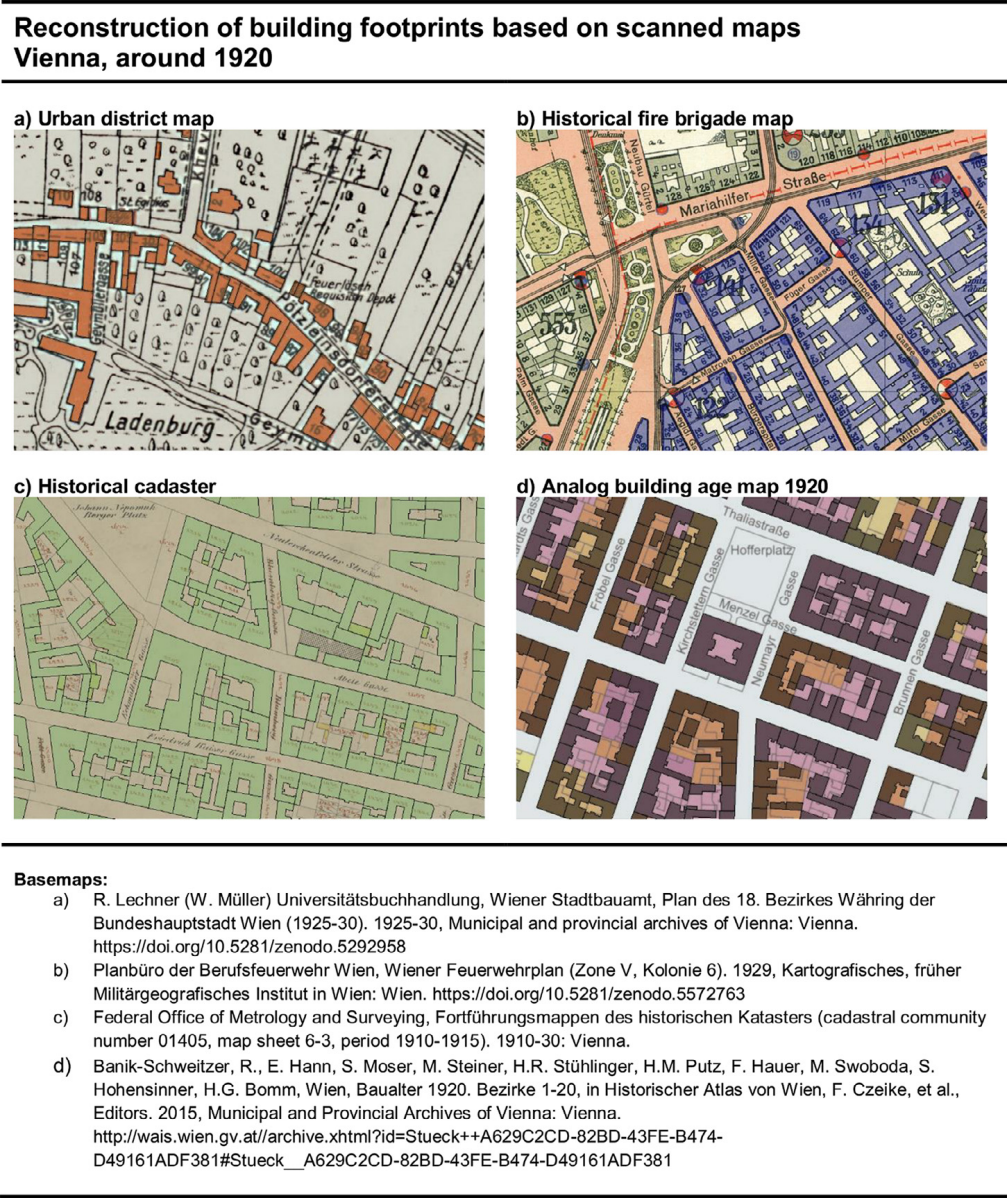
Examples of vectorized building footprints (colored polygons) based on underlying analog building stock maps. Each polygon-feature has a record in the BSM_1920 data field “Polygon.source_id” to document the respective analog map source. Sub-part a: Historic urban district map [11]. Sub-part b: Historical fire brigade map [21]. Sub-part c: Historical cadaster [17, cadastral community number 01405, map sheet 6–3, period 1910–1915] . Sub-part d: Analog building age map 1920 [3].

1. **Retrieving and vectorizing building footprints.** We retrieved the building footprints from three distinctive sources as follows:
  ■ We searched the archives of Viennese institutions (“Federal Office of Metrology and Surveying”, “Viennese library in town hall” and “Municipal and Provincial Archives of Vienna”) and collected 134 historical map sheets from 4 distinctive sources (c.f. Fig. 8): historical cadaster [17], historical urban district maps [4], [5], [6], [7], [8], [9], [10], [11], [12], [13], [14], [15], [16], historical fire brigade maps [18], [19], [20], [21], [22], [23], [24], ABAM_1920 [3]. These documents have 19 different timestamps (1906, 1907, 1908, 1910, 1911, 1912, 1913, 1914, 1915, 1920, 1921, 1922, 1923, 1927, 1928, 1929, 1930, 1935, 1936) and 5 different scales (1:1440, 1:2880, 1:3:500, 1:5000, 1:1000). We used the software AutoCAD MAP 3D 2019 and ArcGIS Desktop 10.7.1 for manual vectorization of building footprints. It is noted that these building footprints can be mapped by using the BSM_1920 dataset and excluding those with “Friedrich Hauer” and “City authority (MA41)” in the data field “Editor”.
  ■ Heritage buildings that remained unchanged since 1920 have been retrieved from today's city map [25]. To reduce the efforts for vectorizing building polygons, on the one hand, and to improve the data quality of polygon geometries, on the other, polygon-features from the following buildings have been retrieved: University of Applied Arts Vienna, Hofburg, parliament, town hall, University of Vienna, hospital Lainz, Schönbrunn palace, university observatory Vienna, Arsenal, Belvedere palace. It is noted that these buildings can be mapped by using the BSM_1920 dataset and selecting “City authority (MA41)” in the data field “Editor”.
  ■ The original version of the ABAM_1920 [3] was based on the compilation of building footprint data from various file formats. Based on a personal piece of information from Mr. Pulz, who was involved in the mapping, these files cannot be recovered and imported with a software tool any more. Only the building footprints of the urban districts 14, 15, 18 and 19 are available in Drawing Interchange File Format (DXF). These files have been used for BSM_1920. It is noted that these buildings can be mapped by using the BSM_1920 dataset and selecting “Friedrich Hauer” in the data field “Editor”.
2. **Georeferencing and merging polygon-features.** Subsequently, we used ArcGIS to import the vectorized building footprints by map sheet and georeferenced them with the spatial adjustment package. The graph of today's street network [28] was used for georeferencing purposes.
  ■ It is noted that we did not correct the position of individual building blocks and buildings within a map sheet area. Although this might improve the location quality of individual buildings, it misaligns the street line and therefore the historical layout of building arrangements. So, the position quality of the building footprints is a trade-off between geodesic accurateness and the esthetic of the city layout.
  ■ It is also noted that the accurateness of georeferencing depended on the hand-drawn footprints and scale of the analog building stock maps. For instance, the historical cadaster at a scale of 1:1,440 was comparably better suited for georeferencing than the analog building age map at a scale of 1:10,000.

After georeferencing the building footprints by map sheet area, the building footprints in the overlapping zones of the map sheets were manually revised by linking, fitting and re-drawing building footprints. The final result of this task is a two-dimensional base map including 88,172 polygon-features within today's spatial city boundaries. It is noted that the adoption of the built-up areas to represent the situation in 1920 (see 3rd step) reduced the number of polygon-features to 81,629, and the cleaning of the dataset (see 4th step) reduced the number to 80,640, which is the final number of polygon-features in BSM_1920.shp.

##### 2.2.2.2 2nd step: Assigning construction periods to building footprints

Basically, we compiled construction dates from 3 different data sources. Multi-assignments of construction dates were resolved by prioritizing the data sources and therefore selecting one construction date for each building footprint. As the construction dates from the 3 data sources have distinctive formats and numbers of unique data records, we converted them into uniform building periods, which are given in the data field “period of construction” or “PoC”.

In detail, the following steps were carried out, whereas the number of unique and assigned construction dates is given in Table 3.

1. **Assigning construction dates (CD)**
  ■ *Data field “CD.abam”.* The ABAM_1920 [3] maps construction periods by color at property scale. We georeferenced the ABAM_1920 district-wise and used the color-recognition features in ArcGIS to assign the color information to polygon-features of BSM_1920. In detail, the ABAM_1920 used 13 colors to indicate 13 different periods of construction. In ArcGIS, we selected 10 training polygons for each color, used the maximum-likelihood-classification algorithm to classify the 13 different colors, and the “tabulate area” function to assign color-based building period information to the building footprints.
  ■ *Data field “CD.bs”.* The year of construction “YoC.1920s” in BS_1920s was assigned to polygon-features in BSM_1920 and recoded in the data field “CD.bs”. The linkage between BS_1920s and BSM_1920 is the building footprint's address. In detail, we carried out the following steps.
    a. *Assigning year 2019 addresses to building footprints.* We used today's address database [26] and related 66,389 address points to polygon-features. In detail, the spatial join function located 59,352 address points within polygon-features and 4,373 address points outside. These points were manually re-located by us to the inside of polygon-features. We then removed duplicated addresses by polygon-feature, which resulted in 39,100 polygon-features with address assignments in the data field “Address.2019”. As the addresses might have changed between 2019 and the 1920s, we manually reviewed and revised the addresses as follows.
    b. *Reviewing and revising 2019 addresses.* We mapped the “Address.2019” records on top of historical maps with building address labels. As we were not able to retrieve map sheets for the entire area of investigation, we introduced the data field “Address_reviewed” to define which “Address.2019” record has been reviewed and not reviewed, respectively. From a city-wide perspective, 61% of polygon-features were reviewed against the address and 39% not. For the 61% of polygon-features, we copied the records from “Addresses.2019” to “Address_revised” and manually added or removed addresses based on the analog address labels. After completing the revisions, we copied the records from “Address_revised” to “Address.1920”. For the 39% of polygon-features that lack historical address labels, we copied the records from “Address.2019” to “Address.1920”. This step produces unvalidated “Address.1920” records.
      Based on this workflow, the “Address.1920” records include 49,586 (61%) reviewed polygon-features with 26,014 address assignments and 23,572 blank records, and 31,054 (39%) of non-reviewed polygon-features with 13,540 address assignments and 17,514 blank records. With respect to the 49,586 polygons under review, 13,108 were subject to manual revision with regard to address additions and/or removals and 36,478 polygons address were confirmed and remained unchanged.
    c. *Assigning construction years to building footprints.* The linkage between BS_1920s and BSM_1920 is based on address records in both datasets. We combined “Street.1920s” and “BN.1920s” from the BS_1920s to the temporary data field “Address.bs” and performed a “join by data field” function with “Address.1920” in BSM_1920. Address matches are recorded with “ID.bs” which corresponds to “ID” in the building schematic. The “YoC.1920s” in BS_1920s corresponds to “CD.bs” in BSM_1920.
  ■ Data field “TP_pub.date_year”. We used analog building stock maps to digitize the building footprints (see 1st step above). Logically, these buildings were constructed before the building stock map was published. We used this logic, extracted the final year from “TP_pub.date” and generated “TP_pub_date_year”. The latter stands for a construction date before the timestamp of the analog building stock map.

2. **Selecting a construction date for each building footprint*****.*** It is inherent to the procedure that the assignment of construction dates from the 3 data sources to the building footprints resulted in multi-assignments per building footprint. With respect to the total number of 80,640 (100%) building footprints, 28,359 (35%) building footprints had one assignment, 35,233 (44%) had two assignments and 17,048 (21%) had three assignments. To select a construction date per building footprint, we prioritized the data sources as given in Table 3. Practically, we created a blank data field “YoC”. Subsequently, we copied priority 1 records to the data field “YoC”, then we copied priority 2 records to blank “YoC” records and, finally, we copied priority 3 records to the “YoC” blanks remaining. The number of assignments by data source is given in Table 3.

3. **Harmonizing construction dates.** The construction dates of the 3 data sources have distinctive formats (periods vs. years) and numbers of unique dates (Table 3). As this broad variety of dates poses a barrier for city and urban district-wise analysis of construction periods, we allocated the construction dates to 13 building periods as follows:
  ■ Ten building periods were taken from ABAM_1920. The ABAM_1920 includes 13 different building periods, of which we selected 7 with a 10-year timespan (“1851–1860”, “1861–1870”, “1871–1880”, “1881–1890”, “1891–1900”, “1901–1910”, “1911–1920”) and 3 with a period before a given year (“≤ 1818”, “≤ 1850”, “≤ 1920”). Two periods (“1819–1876”, “1877–1890”) were not taken because they do not fit into one of the 10-year periods and affect only 396 building footprints. The 396 data records with “1819–1876” and “1877–1890” were allocated to “0–1920”.
  ■ One building period was added (“1921–1930”) to mainly allocate “CD.bs” records because the underlying BS_1920s was published between 1927 and 1930 and therefore includes construction dates of the 1912–1930 period.
  ■ Two building periods (“≤ 1930” “≤ 1940”) were added to allocate construction dates before a given year. These construction dates mainly result from the timestamp of the analog building stock maps.

##### 2.2.2.3 3rd step: Adapting the built-up areas to represent the situation in 1920

A city-wide map was produced in 1904 and 1912 as a compilation of fragmentary and inhomogeneous map sheets from various sources (https://www.geschichtewiki.wien.gv.at/Generalstadtplan) [43]. Even though the scale allows building footprints to be retrieved, the timestamp of the building footprints is unknown and the city-wide map also includes potential future buildings. For these reasons, we retrieved the building footprints directly from historical maps around the year 1920. As these map sheets have 19 different timestamps, ranging from 1906 to 1936, it was challenging to map the building footprints exactly for the year 1920. Due to the variety of timestamps, we approximated the presence of buildings in 1920 based on maps that show built-up areas in 1912 and 1918 as follows:

**1.** **Considering built-up areas of 1918.** The urban sprawl map [31] shows the built-up areas in 1918 and 1957 by color. We georeferenced and vectorized the built-up areas, assigned the color information to each building footprint with the QGIS function “Join Attributes By Location” and removed building footprints within the urban sprawl area between 1918 and 1957. Finally, we created a data field “Location.usm” that records “in” and “out”, depending on the location of the building footprint in or out of the built-up area in 1918.
**2.** **Considering built-up areas of 1912**. The landscape structure map 1912 [30] includes polygon-features for settlement areas that enclose at least one building in 1912. We created a data field “Location.lsm”, used a QGIS function “Join Attributes By Location” to assign “in” or “out”, depending on the location of the building footprint in or out of the built-up area in 1912.

Finally, we defined the data field “Presence.conf”, which confirms the building's presence around the year 1920. A confirmation (data record “yes”) was assigned to buildings inside at least one type of built-up area and to buildings outside of the built-up areas if the publication year (data field “CD.pub.date”) of the analog building stock map (from which we received the building footprint) was before or equal to 1921. A “no” was assigned to all other building footprints that were located outside built-up areas and had a timestamp after 1921. These polygon-features, in total 6543, were removed from the dataset. The resulting dataset with 81,629 polygon-features was the starting point for the clean-up as described in the following step.

##### 2.2.2.4 4th step: Technical clean-up of the building stock map

The manual digitalization of building footprints by 9 different colleagues and the integration of the building footprints in a single geospatial dataset produced partly technical imperfect polygon-features. The cleaning procedure, as presented here, aims to improve the technical quality of polygon-features. The cleaning was performed with PostgreSQL (https://www.postgresql.org/) and the PosGIS extension (https://postgis.net/), with FME Data Integration Platform (https://www.safe.com/fme/) as well as QGIS (https://www.qgis.org). We created a local database to execute advanced queries and to connect and transform datasets. We addressed the following 5 issues: duplicates, multi-polygons, geometry errors, spatial overlaps of polygons and spatial gaps between polygons. For each of the issues, we provide a brief description, the procedure to identify and fix the bugs and summarize the effects of the cleaning procedure.

1. **Duplicates**

Duplicated polygons overestimate the number of building footprints and prevent the automated assignment of data records to building footprints by “spatial join” functions.

We defined 3 options based on 4 criteria to identify duplicates (Table 4). Criteria 1 considers data records in the data fields “ID.bs” and “CD.abam”. If one polygon includes records in both data fields and one polygon in less than 2 data fields, the latter one was removed. Criteria 2 considers the geometry of the polygon-features. Criteria 3 considers the location of polygon-features next to each other. Criteria 4 considers the overlapping area of polygon-features.

**Table 3:** Number of construction date assignments from distinctive data sources. *Note:* “CD” = Construction date; “PoC” = Period of construction; “-“ = not relevant.

				Unique dates		

Data source	Data field	Format of CD	Priority for “PoC”	before CD harmonization	after CD harmonization	Affected polygon-features
Analog building age map 1920	CD.abam	Periods	1	12	10	49,156
Building schematic of Vienna in the late 1920s	CD.bs	Years	2	238	12	3,125
Historic building stock maps	TP_pub.date_year	Years	3	19	3	28,359
Total	PoC	Periods	–	–	13	80,640

**Table 4:** Options and criteria to identify duplicated polygon-features. It is noted that spatial overlaps of less than 95% existed as well, but visual checking has revealed that these polygons are not necessarily duplicates. These overlaps were resolved separately (see point 5). Legend: “-“ = not defined. “*” = The data fields “CD.abam” and “ID.bs” have been considered.

	Criteria			
Option	Data records in 2 data fields *	Geometry	Co-location	Overlap
1	identical	identical	yes	–
2	not identical	identical	yes	≥ 95%
3	not identical	not identical	–	≥ 95%

Option 1 resulted in 314 polygon-features (157 duplicates) and we removed the second polygon-feature of each pair.

Option 2 and 3 have the same threshold for criteria 4. The workflow identified 404 polygons that caused duplicates. These duplicates were cleaned up as follows: First, we automatically removed polygons that met one of the following criteria. In the case of 100% overlap, one polygon was removed. In the case where one polygon had data records in each of the two data fields, and the other had at least one blank record in one of the two data fields, we removed the latter one. This resulted in the removal of 126 polygons and resolved 256 out of 416 overlaps. Second, the remaining 160 overlaps, found in 76 polygon groups, were manually reviewed. The 160 overlaps were resolved be removing 78 polygons (Table 5). In total, we removed 204 polygons to resolve option 2 and 3 duplicates.

**Table 5:** Summary counts for duplicates with overlapping areas of more than 95%. Note: “-“ = not relevant.

Stage	Counted item	Polygon group 1	Polygon group 2	Total
Identification	Polygons per group	3	2	–
	Overlaps per polygon	2	1	–
	Overlaps per group	6	2	–
	Groups	4	196	200
	Polygons	12	392	404
	Overlaps	24	392	416
Automatic correction	Polygons removed	3	123	126
	Overlaps resolved	10	246	256
	Groups resolved	1	123	124
Remaining items for manual review	Overlaps	14	146	160
	Groups	3	73	76
Manuel correction	Polygons removed	5	73	78
	Overlaps resolved	14	146	160
	Groups resolved	3	73	76
Result without duplicates	Polygons	4	196	200
	Overlaps	0	0	0

With respect to option 1–3, 361 (126+78) out of 717 (314+404) polygons were removed to resolve all duplicates.

2. **Multi-polygons**

Multi-polygons are features that include more than one polygon. They prevent the building-specific assignment of data such as addresses.

We used spatial queries and identified 57 multi-polygons before and 156 multi-polygons after fixing the geometry errors.

We used QGIS's “Multipart to Singleparts” tool to convert 156 multi-part features into 315 single-part features. Next, we removed 255 single-part features automatically because the size was so small that they were not intentionally digitized from the analog building stock maps. The remaining 60 single-part features, covered by 30 multi-part features, needed manual review on the map. In 18 out of 30 cases, we confirmed the presence of the single-part feature, which was due to the underlying footprints in the analog building stock map. In 12 out of 30 cases, the single-part features were part of existing buildings and we merged them with other existing polygons.

3. **Invalid geometries**

Invalid geometries are technical flaws of features.

We used the QGIS tools “check validity” and “check geometries“ and identified 506 errors in 4 categories (Table 6).

We corrected the errors in three steps. First, we used the same QGIS tools that identified the errors to correct them automatically. Second, we filtered the affected polygons and visually detected 15 polygons with flawed geometry changes. For these 15 polygons, we manually corrected 9 self-intersections and 6 duplicated rings. Third, the remaining 491 (506–15) polygons were not affected by geometry changes and we accepted the automatic corrections.

4. **Spatial overlaps of polygons**

Overlapping polygons occurred because of imprecise vectorization of the building footprints from underlying analog maps. The overlaps potentially prevent the assignment of data records by spatial joins and overestimate the area of building footprints.

To identify overlapping polygons, we defined a 2 m^2^ threshold and distinguished between overlaps below and above the threshold, respectively. We used advanced spatial queries and identified 19,379 overlaps in total, of which 18,240 are below and 1139 are beyond the 2 m^2^ threshold.

We corrected the overlaps as follows: First, the 18,240 overlaps below the threshold were automatically removed by using the FME Data Integration Platform and applying the “AreaGapAndOverlapCleaner” transformer. This transformer “repairs area topologies by resolving gaps and overlaps between adjacent areas” (https://docs.safe.com). Second, the 1,139 overlaps beyond the threshold affected 1,078 polygons. We applied 5 different measures to resolve these overlaps manually in QGIS (Table 7).

5. **Spatial gaps between polygons**

**Table 6:** Summary of geometry errors and corrective measures.

Error category	Identified	Corrected automatically	Corrected manually by adopting the geometry
duplicated rings	6	4	6
ring self-intersections	45	45	0
self-intersections	420	411	9
too few points in geometry component	35	35	0

**Table 7:** Manual measures to resolve overlaps.

Measure	N° polygons
polygon removed	354
geometry adapted	221
no measure	304
merging of overlapping polygons	199

Spatial gaps occurred owing to the close location of buildings, whereas adjoined polygon segments are not positionally congruent. The gaps were caused by imprecise vectorization of building footprints or, more precisely, by faulty snapping of vertices and segments. Most of these gaps are quite small and can only be visually detected by zooming into the map. These gaps did not cause technical problems in assigning data to polygons. It's more a question of optical quality and accuracy when it comes to keeping or closing the gaps.

**Fig. 9:**
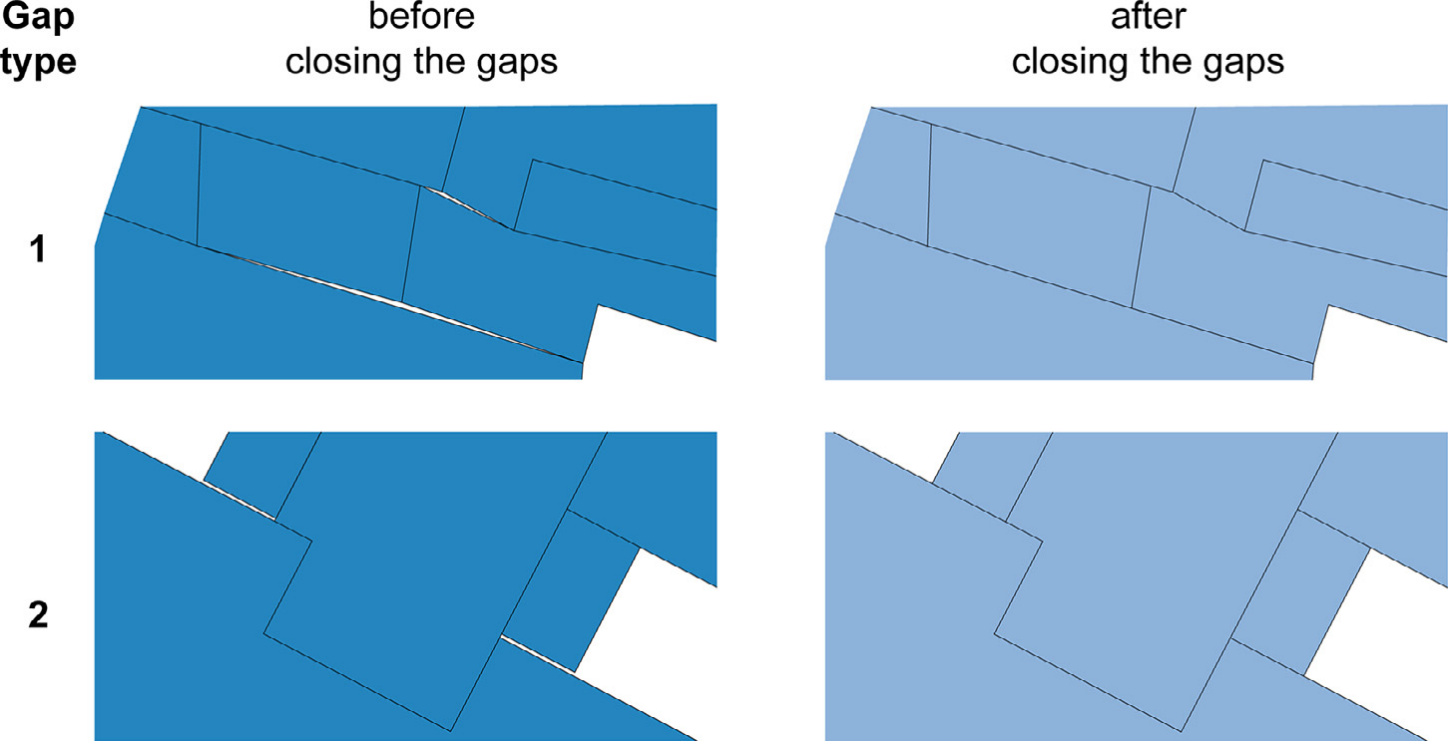
Example for gap types 1 and 2, before and after closing the spatial gaps between polygons.

The manual review of polygons on the map revealed two distinctive gap types. One type that is completely locked in by polygon segments and another that is surrounded by polygon elements close to but not completely locked in by polygon segments (Fig. 9).

Before we started to identify and close the gaps, we defined the following quality criteria.

■ Intentional gaps (e.g. passageways, light shafts) should remain but unintentional gaps should be resolved.
■ Buildings standing next to each other should have adjoining polygon segments without any gaps between.
■ The basal geometry of polygons should remain as far as possible. In other words, the adoption of polygon geometries should not result in invalid geometry artifacts.
■ Polygons that are completely surrounded by another polygon without overlapping it should remain.

The gaps were found in a vast number of different polygon settings and we learned that we cannot apply a single tool to achieve the aforementioned quality criteria. So, we experimented with the FME Data Integration Platform (Snapper transformer, AreaGapAndOverlapCleaner transformer), QGIS (Snap geometries to layer, Check geometries) and GRASS GIS (v.clean - snap tool, v.in.ogr - snap on import) to identify a suitable combination of tools and settings. For instance, we defined the maximum distance for the gap identification with 0.01, 0.03, 0.31 and 1 m, applied the QGIS function “Snap geometries to layer” with 8 different options, and plotted the number of affected polygons. For one of these options, to give an example, we found that 9,561, 15,573, 21,819 and about 23,000 polygons are affected by the various distance settings. Based on that finding, we concluded that the majority of gaps are covered by a distance on a single-digit decimetre scale. We also noticed that 101 polygon geometries were completely destructed, independently of the tool and its settings, and we manually removed 68 and merged 33 with others. In the experimental phase, we learned that a semi-automated procedure that combines automatic corrections, visual review of the effects on the map and refined tool settings is beneficial to meet the aforementioned quality criteria. It is noted that these steps were completed after we were personally satisfied with the results because we didn't find a quantitative measure for defining and assessing quality criteria to resolve gaps in manually digitized building stock maps.

Based on the experiences in the experimental phase, we finally carried out the following steps:

1. We set the distance at 0.321 m and applied the QGIS vector geometry function “Snap geometries to layer” and selected the parameter “behavior” with the option “Prefer closest points, don't insert new vertices”. The selection of the threshold was an iterative process in which we varied the mean value, applied the snapping function, and visually evaluated the effects on adopted geometries. We finally defined 0.356 m as a threshold, which slightly adapted the geometries and retained small-scale elements such as light shafts, thoroughfares between buildings and garden walls unchanged. Overall, the procedure snapped vertices of a polygon to the closest point of another polygon. This step was needed to realign polygon segments in the next step.
2. To realign polygon segments next to each other, we limited the accepted distance to 0.3 m. This value was used for applying the QGIS vector geometry function “Snap geometries to layer” and selected the parameter “behavior” with option “Prefer aligning nodes, don't insert new vertices”. Subsequently, we manually screened adopted polygon geometries and recovered the original version if the adopted geometries contradicted the building footprint in the analog building stock map.
3. Steps 1 and 2 generated minor polygon overlaps. These overlaps were removed by FME transformer “AreaGapAndOverlapCleaner” with a tolerance value lower than 0.0001 m^2^.
4. We used 0.15 m distance and applied the FME transformer “Snapper”. Subsequently, we manually screened adopted polygon geometries and recovered the original version if the adopted geometries contradicted the building footprint in the analog building stock map.
5. We used the QGIS function “check geometries” to remove gaps within polygons smaller than 0.8 m^2^ and also removed more than 8,000 duplicated nodes of unique polygons.

In summary, the manual digitation of building footprints and their integration into a single geospatial dataset produced technical flaws that were addressed by resolving 5 issues: duplicates, multi-polygons, invalid geometries, spatial overlaps of polygons and spatial gaps between polygons. Resolving the technical flaws was a semi-automated procedure. A statistical summary of the clean-up effects is given in Table 8. To detect the number of polygon-features with geometry changes, we applied the QGIS function “Detect dataset changes". This function compares “two vector layers, and determines which features are unchanged, added or deleted between the two” (https://www.qgis.org). Based on this analysis and with respect to the final number of 80,640 polygon-features, 80,064 share the same identification, of which 36,078 remained unchanged and 44,536 underwent geometrical changes. The geometrical changes were affected by 68,379 interventions (e.g. adding, removing and re-relocating vertices), of which 32,978 resulted area extensions and 35,401 resulted in area reductions.

**Table 8:** Statistical summary of the clean-up process. *Notes:* “1” = Area extension of existing polygon-features; “2” = Area additions because new polygon-features were added; “3” = Area reduction of existing polygon-features; “4” = Area removal because polygon-features were entirely removed.

	Before cleaning	Added	Removed	After cleaning
Total area [m^2^]	22,982,900	26,000^1^		
5,200^2^	17,600^3^			
206,800^4^	22,789,700			
Mean area per polygon-feature [m^2^]	281.56	0.79	0.50	282.61
Minimum area per polygon-feature [m^2^]	0.00009	2 × 10–23	2 × 10–23	0.14148
Maximum area per polygon-feature [m^2^]	29,769	2779	855	29,770
Number of polygon-features	81,629	26	1015	80,640

#### 2.2.3 Mapping the scope of the analog building age map

**Fig. 10:**
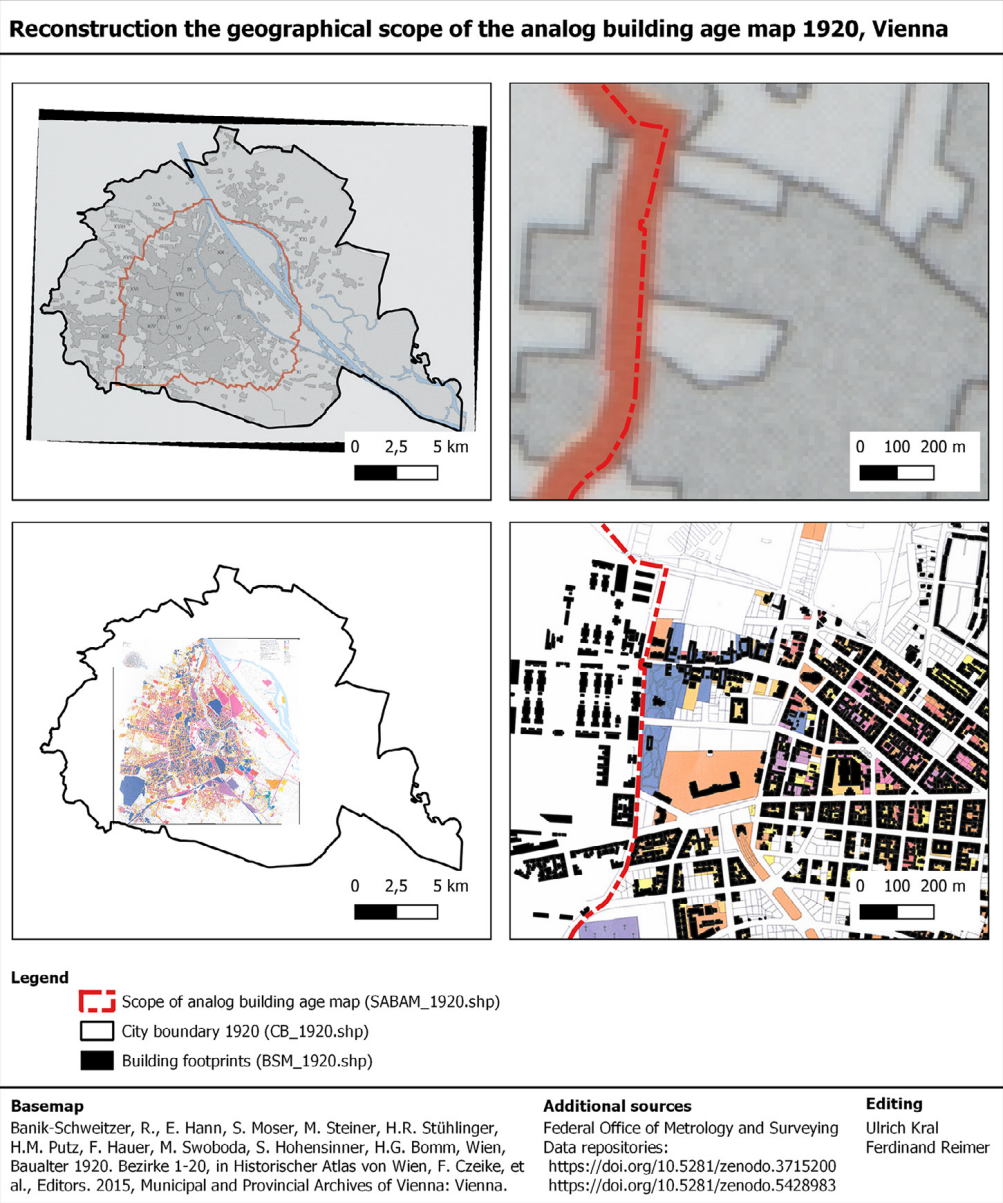
Reconstruction the geographical scope of the analog building age map 1920. *Note:* For interpretation of the references to color in this figure legend, the reader is referred to the web version of this article.

The ABAM_1920 covers most of the city area at this time. However, it excludes the city's outskirts in 1920 as well as areas that were incorporated to the city between 1920 and 2020 (Fig. 10). To compare the geographical coverage of the ABAM_1920 with those from the BAM_1920, we reconstructed the geographical scope of the ABAM_1920 as followed.

1. The overview map of the ABAM_1920, which was previously georeferenced by Kral et al. [50], (Fig. 4), shows an orange colored line, which is an overview on the geographical coverage of the dataset. We used a line-feature and manually vectorized the orange line. The large scale and low resolution of the overview map does not allow to reconstruct the course of the boundary on building level. So, the reconstructed line is an auxiliary line that is refined in the next step.
2. A more precise mapping was done by using the building age map (BAM_1920) on the one hand and the georeferenced analog building age map 1920 (ABAM_1920) on the other. Both maps show the building footprints. The ABAM_1920

#### 2.2.4 Assigning external data records

We used QGIS functionality for joining attributes of polygon-features by their location to generate the data records for the four data fields as listed in Table 9.

**Table 9:** Spatial layer join. Notes: “BSM_1920” = Building stock map 1920; “CB_1920” = City boundary 1920, “UDB_1920” = Urban district boundaries 1920”; “SABAM_1920” = Scope of analog building age map 1920.

Data fields in target layer BSM_1920	Source layer (file name)	Source layer (Refs.)
Location.city_limit_1920	CB_1920.shp	[2]
UD.1920	UDB_1920.shp	[2]
UD.2020	Wien_gstgenau.shp	[27]
Location.ABAM_1920	SABAM_1920.shp	[49]

### 2.3 Data output validation

This section validates the quality of datasets and is grouped into four parts. First, the technical quality check of data records in BSM_1920. Second, the technical quality check of polygon-features in BSM_1920. Third, external datasets are used to check the plausibility of the building counts based on the building footprint areas BSM_1920 and the presence of building footprints around 1920 in BSM_1920. The assignment of construction years from BS_1920s to BSM_1920 is evaluated as well as the fit of construction years within the period of construction. Fourth, areas of quality improvements are identified.

#### 2.3.1 Technical quality check of data records

The BSM_1920 is used to assess the consistency of data records in terms of format and completeness. Basically, data records of 8 key data fields are firstly grouped by urban district (“UD.1920” and/or “UD.2020”), secondly grouped by their unique data records (e.g. “in” and “out” in data field “Location.city_limit_1920”) by alternative categories, thirdly, summarized by building footprint area (“Area”) and fourthly, plotted in Figs. 4 and 5. The figures demonstrate the completeness of data records because the sum of all bars per plot equals the total building footprint area of 2279 ha. The figures also demonstrate the consistency of the data format because the grouping allocates each data record to a predefined category. It is noted that the code availability section presents a usage code to generate Figs. 4 and 5 based on the recorded datasets.

#### 2.3.2 Technical quality check of polygon-features

The BSM_1920 is used for assessing the topology and geometry of polygon-features as follow:

■ We used the QGIS function “Topology checker” and selected the rules “must not have duplicates”, “must not have invalid geometries”, “must not overlap” and “must not have multiple-part geometries”. Based on these settings, the function didn't detect any errors.
■ We used the QGIS function “Geometry check” and selected the geometry validity criteria “self intersections”, “duplicate nodes”, “self contacts” and “polygon with less than 3 nodes”. Based on these settings, the function didn't detect any errors.

#### 2.3.3 Content-related plausibility check by external data sources

##### 2.3.3.1 Building counts by urban district

We used the parameter “building counts by urban district” to validate the BSM_1920 with the building counts from the statistical yearbook of 1929 (SY_1929) with records for urban district areas in 1923 [51] and the statistical yearbook of 1934 (SY_1934) [52]. The basic challenge was to identify comparable numbers.

■ The SY_1929 and SY_1934 recorded the number of “houses” based on censuses. A ‘house’ is a residential building as defined in the Census Act at 29 March 1869 (http://alex.onb.ac.at/cgi-content/alex?aid=rgb&datum=1869&size=45&page=341). Consequently, adjacent, commercial and public buildings were not recorded. In other words, the statistic underestimates the total number of buildings at this time.
■ The BSM_1920 covers polygon-features for all buildings regardless of their use and size. To approximate the number of “houses”, we estimated the number of polygon-features with a grated approach that sets the area equal or larger than 80, 100 and 120 m^2^, respectively. The mapping of the buildings less than 80, 100 and 120 m^2^ shows a tendency for excluding buildings additions (e.g. external stairways), adjacent buildings and other small-sized buildings. It does not exclude commercial and public buildings larger than 80, 100 and 120 m^2^. So, the graded approach reduces the number of polygons to approximately the number of houses but does not exclude all non-residential buildings. Consequently, the estimated number goes beyond the ‘housing’ counts as defined by the census.

**Fig. 11:**
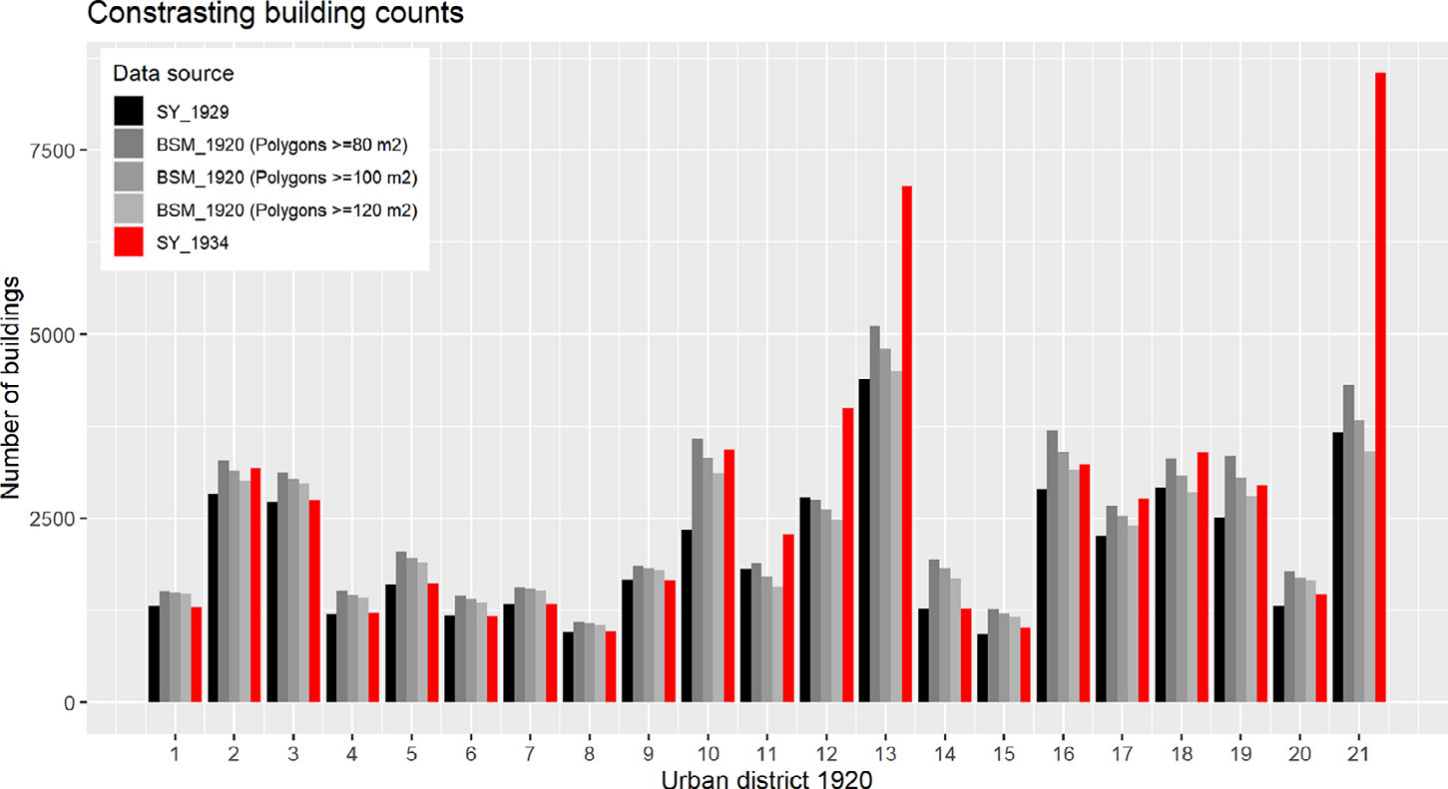
Contrasting building counts. The building stock map (1920s) considers polygons as buildings if the area is larger than or equal to 80, 100 and 120 m^2^. Notes: “SY_1923” = Statistical yearbook 1929 [51] with building counts from 1926. “BSM_1920” = Building stock map 1912. “SY_1934” = Statistical yearbook 1934 [52]. Note: For interpretation of the references to color in this figure legend, the reader is referred to the web version of this article.
■ The building counts of BSM_1920 are by tendency larger than SY_1929 records for 1923. This is because the numbers include commercial and public buildings as well as side-buildings larger or equal to 80, 100 and 120 m^2^, respectively.
■ The aerial grading of the polygon-features shows the influence of small-sized buildings in relation to larger-sized buildings exemplified, for instance, with the inner urban district 1–9 with large-sized residential and representative buildings, on the one hand, and the greener and less dense populated outer district 21 with more small-sized buildings, on the other.

The contrast of the building counts between BSM_1920, SY_1929 and SY_1934 is shown in Fig. 11. The figure allows the following conclusions.

**Fig. 12:**
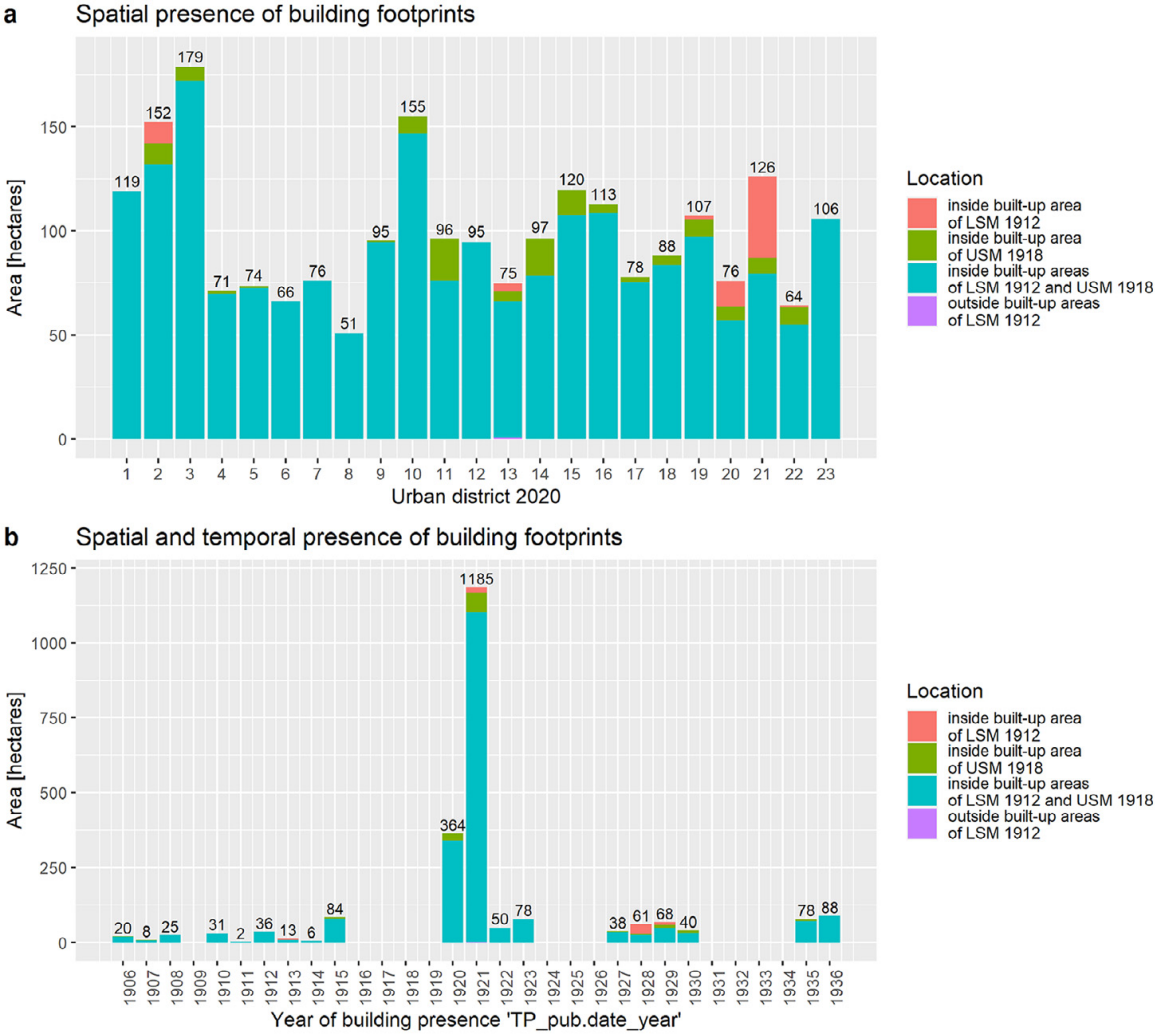
Presence of building footprints in spatial and temporal perspective. Sub-part a: Spatial presence of building footprints inside or outside of built-up areas in 1912/18. Sub-part b: Spatial presence of building footprints inside or outside of built-up areas in 1912/18 plotted against the temporal presence as retrieved from analog building stock maps. Note 1: “LSM 1912” = Landscape structure map with built-up areas in 1912; “USM 1918” = Urban sprawl map with built-up areas in 1918. Note 2: For interpretation of the references to color in this figure legend, the reader is referred to the web version of this article.

■ The building counts of BSM_1920 are closer to SY_1929 records for 1923 than to SY_1934 records. This might be because 84% of the area has a timestamp before 1923 and only 16% between 1927 and 1936 (Fig. 12b). We conclude that the building stock map in its current version represents the situation in the early 1920s and does not cover urban sprawl areas from the late 1920s and early 1930s, especially in districts 13 and 21. An exception is district 10, which shows building counts close to the year 1934. We didn't have a clear explanation for this, but it might be because 50% of the area has a timestamp of 1935, 41% a timestamp of 1921 and 9% a timestamp before 1911. The urban sprawl map, which has been used to define built-up areas in 1918 and to remove building footprints out of this built-up area, does not indicate the building density. So, it might be that rapid urban development in this period condensed the building density, but we are were not able to identify unbuilt plots within the built-area. All in all, the limitation of buildings to the year 1920 in areas of rapid urban development challenged the mapping in the 10th district.

This validation step contrasted building counts on an urban district level. Based on the validation results, we feel that the digitized buildings are plausible for comparative urban district analysis. This validation approach does not assess the plausibility of each individual building, either in terms of presence in the early 1920s or in terms of assigned information (e.g. area of building footprint, exact location, building period). Researchers who are interested in the historical situation of specific buildings need to retrieve site-specific historical data to get valid information.

##### 2.3.3.2 Building footprint presence around 1920

We made plausible the presence of the building footprints around 1920 by assessing the localization of the building footprints inside and outside of built-up areas at this time (spatial presence) as well based on the publication year of the analog building stock map from which we retrieved the building footprints (temporal presence).

■ *Spatial presence.* From a city-wide perspective, 91.4% of the building footprint is located in the built-up areas of 1912 and 1918, 5.6% is located in the built-up area 1918 only, 3.0% is located in the built-up area 1912 only and less than 0.05% is located outside the built-up areas with a timestamp before or equal to 1921. The data on an urban district level are presented in Fig. 12a. The validation of the building presence around 1920 is more an approximation of the real situation than a timewise exact mapping of individual buildings. Although more than 99.95% of building areas are located within the built-up areas of 1912/18, it might be that individual buildings have been constructed at a later stage (Fig. 12b).
■ *Temporal presence.* Based on the publication year of the analog building stock maps, we identified 84% of building footprint areas with a presence before 1923 and 16% between 1927 and 1936 (Fig. 12b). Latter ones were potentially also available before 1927, but we didn't find direct evidence due to a lack of historical maps at this time. Urban district analysis reveals that buildings with a confirmed presence later than 1923 can be found in districts 2, 5, 6, 10 and 21–22. As the districts 5, 6 already had a high building density in 1912 [43], we feel that the recorded buildings were present around 1920 to a large extent. In contrast, the urban districts 2, 10, 21 and 22 were not fully developed at this time. However, the recorded building counts for districts 2 and 21, 22 (Fig. 11) are closer to the statistical yearbook 1923 records than those in the 10th urban district. We conclude that the number of buildings as well as the building footprint areas in the 10th district are overestimated. Experts wishing for a more realistic picture for the 10th district might profit from searching and retrieving additional historical maps with a timestamp around 1920.

##### 2.3.3.3 Construction years

**Fig. 13:**
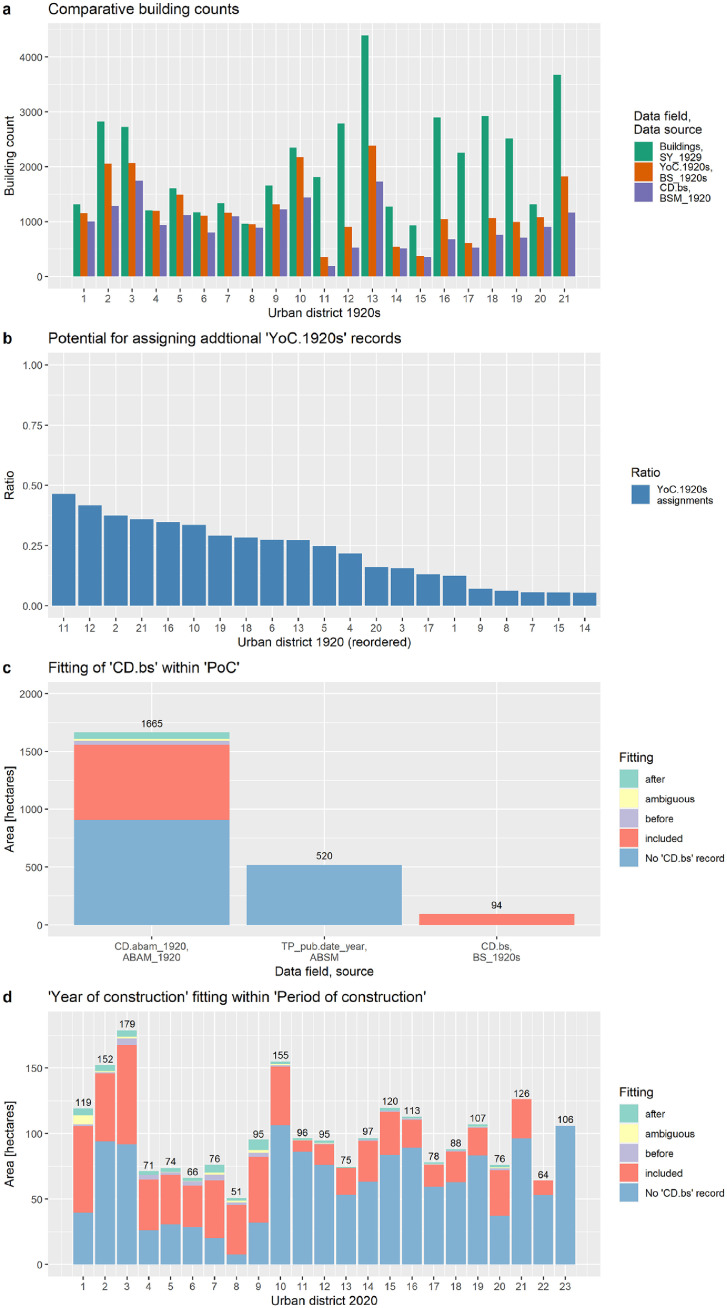
Validating construction years and periods. Sub-part a: Comparative building counts based on the number of buildings in the SY_1923 dataset, the number of “YoC.1920s” records in the BS_1920s dataset and the number of “CD.bs” records in the BSM_1920s dataset. Sub-part b: Potential for assigning additional ‘YoC.1920s” records to buildings in the building stock map. Sub-part c: Fit of “CD.bs” in “PoC” by data source. Sub-part d: Fitting of “CD.bs” in “PoC” by urban district. Note: For interpretation of the references to color in this figure legend, the reader is referred to the web version of this article.
■ *Temporal presence:* The temporal presence between 1927 and 1936 was confirmed for 16% (373 ha) of the total building footprint area (2,279 ha). These areas are mainly located in districts 2, 5, 6, 10, 21 and 22. Even though these buildings are located in the built-up area of 1912 and 1918, their presence in 1920 remains unvalidated. This is because the built-up area are single polygons without differentiation between built and unbuilt properties. To improve the data quality, we suggest retrieving additional historical building stock maps and, based on that, revising the temporal presence as well as the building geometry as required. We expect that the dense build-up and fully developed urban districts 2, 5 and 6 are less affected by potential revisions compared to urban districts of rapid urban development at this time, such as districts 10, 21 and 22. However, retrieving historical maps for the aforementioned districts is challenged by the fact that map sheets of the historical cadaster, the fire brigade maps and the urban district maps do not cover the entire city around the year 1920. Nevertheless, intensifying the historical investigation in Viennese archives is needed to potentially find appropriate maps.

The year of construction (“CD.bs”) records in BSM_1920 were retrieved from “YoC.1920s” records in BS_1920s [29]. This validation step analyses the number of assignments from a BS_1920s and SY_1929 perspective [51]. The following numbers give a city-wide overview and Fig. 13a provides insight on an urban district level.

■ BS_1920s perspective: The BS_1920s includes 25,859 (100%) entries with records in “YoC.1920”, “STR.1920s” and “BN.1920”. The latter two are needed to join the BS_1920s and BSM_1920 dataset. The workflow captured 19,631 (76%) “YoC.1920s” records and assigned them to buildings in BSM_1920. In other words, the remaining potential for assignments is 24%.
■ SY_1923 perspective: The SY_1929 records 43,910 residential buildings for 1923. So, the 19,631 “YoC.1920s” assignments to BSM_1920 cover 45% of all residential buildings. Considering the total number of 25,859 available “YoC.1920s” records in the BS_1920s, the maximum rate of potential assignments is 59% with respect to all residential buildings in the 1920s.

In conclusion, the assignment of construction years is limited by the data availability in the BS_1920s, the scope of the BS_1920s (which covers 94% of all buildings footprints) and the data quality of the address data in BS_1920s and BSM_1920. The workflow assigned “YoC” records from the BS_1920 to about 45% of all residential buildings in the BSM_1920. The maximum ratio of about 59% might be achieved by improving the data quality of “Address.1920s” records in BSM_1920. An efficient workflow for address data quality improvement should focus on the potential of additional “YoC.1920s” assignments. We estimated the potential by defining the ratio by urban district as given in Eq. (1). With respect to urban district level, the highest potential is in the 11st district and the lowest potential in the 14th district (Fig. 13b).

$$\begin{array}{c} \text{Ratio}\;\;\;\;\;\;\;\;\;\;\;\;\;\;\;\;\;\;\;\;\;\;\;\;\;\;\;\;\;\;\;\;\;\;\;\;\;\;\;\;\;\;\\ =\;\frac{\text{Number}\;\text{of}\;\;``\text{YoC}.1920s"\;\;\text{records}\;\text{in}\;\text{BS}\_1920s\;-\;\text{Number}\;\text{of}\;``\text{CD}.\text{bs}"\;\text{records}\;\text{in}\;\text{BSM}\_1920}{\text{Number}\;\text{of}\;``\text{YoC}.1920s"\;\;\text{records}\;\;\text{in}\;\text{BS}\_1920s} \end{array}$$

Eq. (1): Ratio to assess the potential of additional address validation steps.

##### 2.3.3.4 Building periods

The period of construction “PoC” records were retrieved and compiled from 3 different sources, whereby 73% of all building footprint areas received the “PoC” from the building periods of the ABAM_1920 [3], 23% from the publication year of the analog building stock maps [4], [5], [6], [7], [8], [9], [10], [11], [12], [13], [14], [15], [16], [17], [18], [19], [20], [21], [22], [23], [24], and 4% from the BS_1920s [29] (Fig. 13c). Data on urban district level are plotted in Fig. 13d. In general, the ratios are driven by prioritizing the data sources for the compilation and assignment of periods of construction “PoC” (see Section 2.2.2.2). This validation step tests if the construction years “CD.bs” fit within the period of construction “PoC”. It is noted that we excluded those building footprints (94 ha, 4% of total footprint areas) that retrieved the “PoC” information directly from the BS_1920s, which is the data source for validation. The remaining building footprint areas of 2,185 ha (96%) were taken into consideration as follows:

■ 62.5% of the footprint areas have a blank “CD.bs” record. A blank record can occur for two reasons. First, missing “Address.1920” records, which prevent the linkage to BS_1920s and therefore retrieve “YoC.1920s” records. Second, non-verified and therefore potentially wrong “Address.1920” records, which also prevent the linkage to BS_1920s and retrieve “YoC.1920s” records. Both the missing and non-verified “Address.1920” data could be addressed by making data quality improvements in the future. Such improvements could focus on the complete reconstruction and validation of historical addresses. Finally, it is noted that the workflow assigned a “YoC.1920s” record to 45% of the buildings, whereas the maximum achievable assignment rate is 59% (see Section 2.2.2.2).
■ 32.8% have a construction year “CD.bs” within the period of construction “PoC”. This confirms two things. First, the assignment of building periods in the ABAM_1920 are correct for these building footprints areas. Second, the transfer of the building period information from the ABAM_1920 to BSM_1920 is correct.
■ 3.9% of building footprint areas have a “CD.bs” before or after the “PoC”. The lack of fit might be for the following reasons. First, the “CD.bs” and “CD.abam” records in the input data sources might be incorrect. Second, the assignment of “CD.bs” from the BS_1920s failed. Third, the assignment of “CD.abam” from the ABAM_1920 failed.
■ 0.8% of the building footprint area is classified as “ambiguous” because the “CD.bs” record includes multiple single years that fit into distinctive periods of construction “PoC”. So, the “CD.bs” assignment is ambiguous in view of the defined building period classes.

This validation step tested the fit of “year of construction” between the start and end of the “period of construction”. If the building footprint sample is limited to those that have a “CD.bs” and “PoC” record (854 ha, 39% of the total building footprint area), we found an 87% fit. The remaining 13% have a “CD.bs” record either before/after the start/end of the “PoC” or the fit is ambiguous because of multiple “CD.bs” assignments. It is noted that we didn't run a systematic data analysis to identify the reasons for the non-fitting years within periods or develop a tailored strategy for resolving or explaining the non-fitting construction years. But, we recommend measures to improve the data quality in the next section.

### 2.4 Conclusions in view of data quality improvements

The reconstruction of the historical building stocks and their respective ages was a time-consuming effort. We experienced that the efforts are influenced by the aspiration to achieve a certain level of data quality. The data quality involves, among other areas, the coverage of buildings within the city limits 2020, the accurateness of building geometries and their localization, the temporal presence of the building footprints for 1920 and the coverage, accuracy and plausibility of the buildings’ construction dates. All of these aspects are affected by the availability of historical information and the capability to convert them into machine-readable data formats. Personal discussions with data providers, among the author team and experts in the field of urban history, cultural heritage and urban planning have shown that the level of data quality is primarily a matter of the rationale for using the data. Our rationale was to generate a dataset for a given year as a starting point to analyze the spatio-temporal development of the building stock in the future. The spatial dimension addresses the urban district level and the temporal dimension deals with decades. In this sense, the data quality was validated on the urban district level for the time around 1920 but not for each individual building exactly for the year 1920. Experts who are interested in validated data of individual buildings have to retrieve additional historical information and integrate them into the datasets. The datasets [49], which are related to this article, are a starting point to improve the data quality and potentially supplement additional attributes.

Based on our experience during the development of the datasets, we identified areas of potential data quality improvements. It is noted that these areas, as listed below, reflect our personal aspirations and do not necessarily cover the aspirations of other experts in the interdisciplinary environment of building stock research. Nevertheless, the areas listed here should inspire experts to use and develop the datasets further.

■ *Coverage of buildings within the city limits 2020.* The BSM_1920 covers the city area in 2020, but excludes 1.3% of the total settlement area of 1920. The excluded area can be found on the outskirts of today's 13th district in Fig. 2. To record the buildings from the currently excluded settlement areas, historical building stock maps need to be retrieved and building footprints need to be vectorized and georeferenced.
■ *Geometry of building footprints.* The building footprints were manually constructed by going over analog building stock maps. The manual reconstruction potentially includes geometry flaws such as imprecise reconstruction of parallel and orthogonal polygon segments. Data quality of geometries can be improved by identifying buildings that remain unchanged between 1920 and 2020 and incorporating the respective polygon-features from today's geospatial building dataset [25] to the BSM_1920. We followed this approach for 10 historical buildings (see Section 2.2.2.1, but there might be additional building footprints that profit from this approach.
■ *Localization of building footprints.* We digitized the building footprints by going over non-georeferenced analog building stock maps. Then the polygon-features, covering the area of a map sheet, were georeferenced based on historical street courses and today's street graph [28]. We experienced that spatial referencing by map sheet is challenged by linking polygon-features of side by side map sheets, in areas of wide-meshed street networks and by polygon-features that were digitized based on large-scale analog maps (1:5000 and 1:10,000). By tendency, spatial referencing in dense built-up areas is more accurate than in loosely built-up areas. We feel that the localization of building footprints can be improved by re-referencing the building footprints by settlement areas instead of by map sheets. In particular, the settlement areas beyond the city boundaries of 1920 might profit from this approach because the rural settlement structure covers mainly well delaminated villages. In these cases, the referencing should be based on the building polygon-features of today's city map [25] instead of today's street graph [28].
■ *Construction dates.* Three distinctive datasets with construction dates were linked with the BSM_1920, a single construction data was selected for each building footprint and the selected constructed dates were categorized into 13 buildings periods (see Section 2.2.2.2). With respect to the total building footprint area of 2,279 ha, 63% were categorized by a 10-year period and 27% have a construction date before a given year (Fig. 5a). We see a potential to re-categorize buildings, which are part of the 27% share, into a 10-year period and therefore provide a higher temporal resolution for the construction date by the following two actions. First, the assignment ratio of construction years from the BS_1920s dataset relative to BSM_1920 could be increased by improving the “Address.1920” records. Practically, polygons with “no” records in the data field “Address_reviewed” could be selected and subject to review and corrections based on historical evidence given by address labels in historical building stock maps. Based on this action, the assignment rate (number of buildings with a “CD.bs” record relative to the total number of buildings) could be lifted from currently 45% towards 59%. The highest potential for data quality improvements, relative to the number of buildings per urban district, is found in district 14, followed by 12 and 2 (Fig. 13b). Second, the aforementioned ratio of maximum assignments is limited to 59% because the BS_1920s covers construction year records for 59% of the buildings at this time. Ratios beyond 59% can be achieved by retrieving the construction years and periods, respectively, from alternative historical sources such as the general city map 1912 [43], historical documentation on the development of settlement areas or building-specific construction plans. Construction plans, archived by the Viennese building authority (Municipal Department 37: Building Inspection), are available for historical buildings that are still standing today. Nevertheless, retrieving construction plans and adding more construction year records to BSM_1920 is time-consuming and should be balanced against the overall purpose of the dataset.

## 3 Code Availability and Usage Notes

The corresponding GitHub repository “building.map_1920” [53] includes a “Readme.md” file with further guidance and instructions, a “Codebook.pdf” file to specify the data format, the data fields and comments on the data fields of the datasets BSM_1920.shp and the attribute table BSM_1920_attribute_table.csv, a “usage code” to reproduce, as for instance, the figures of this article, and a “background data” file.

The SHP and CSV files can be imported and processed with QGIS (https://www.qgis.org), ArcGIS (https://www.arcgis.com), PostGIS (https://postgis.net/), the R Project for Statistical Computing (https://www.r-project.org/) and any other tools that enable geospatial data processing.

## Ethics Statement

We declare compliance with ethical standards for scientific publishing. This work is not based on animal and human studies.

## CRediT authorship contribution statement

**Ferdinand Reimer:** Methodology, Data curation, Formal analysis, Investigation, Visualization, Writing – original draft. **Ulrich Kral:** Conceptualization, Methodology, Data curation, Investigation, Formal analysis, Validation, Visualization, Writing – original draft, Funding acquisition. **Emre Can Sönmez:** Investigation, Writing – original draft. **Friedrich Hauer:** Investigation, Data curation, Writing – review & editing. **Severin Hohensinner:** Investigation, Data curation, Writing – review & editing. **Hannah Wolfinger:** Investigation, Writing – review & editing. **Klara Stuppacher:** Investigation, Writing – review & editing. **Andreas Danzinger:** Investigation, Writing – review & editing. **Ingeborg Hengl:** . **Lupina Prospero:** Investigation, Writing – review & editing. **Sarah Prunner:** Investigation, Writing – review & editing. **Helmut Rechberger:** Supervision, Writing – review & editing.

## Declaration of Competing Interest

The authors declare that they have no known competing financial interests or personal relationships which have or could be perceived to have influenced the work reported in this article.
